# Enzymatic Methods for the Site-Specific Radiolabeling of Targeting Proteins

**DOI:** 10.3390/molecules26123492

**Published:** 2021-06-08

**Authors:** Cristina Bolzati, Barbara Spolaore

**Affiliations:** 1 Institute of Condensed Matter Chemistry and Technologies for Energy ICMATE-CNR, Corso Stati Uniti, 4, I-35127 Padova, Italy; 2 Department of Pharmaceutical and Pharmacological Sciences, University of Padua, Via Marzolo, 5, I-35131 Padova, Italy; 3 CRIBI Biotechnology Center, University of Padua, Viale G. Colombo, 3, I-35131 Padova, Italy

**Keywords:** radioimmunoconjugates, transglutaminase, sortase, lipoic acid ligase, galactosyltransferase, PET, SPECT, mAb, nanobody, affibody

## Abstract

Site-specific conjugation of proteins is currently required to produce homogenous derivatives for medicine applications. Proteins derivatized at specific positions of the polypeptide chain can actually show higher stability, superior pharmacokinetics, and activity in vivo, as compared with conjugates modified at heterogeneous sites. Moreover, they can be better characterized regarding the composition of the derivatization sites as well as the conformational and activity properties. To this aim, several site-specific derivatization approaches have been developed. Among these, enzymes are powerful tools that efficiently allow the generation of homogenous protein–drug conjugates under physiological conditions, thus preserving their native structure and activity. This review will summarize the progress made over the last decade on the use of enzymatic-based methodologies for the production of site-specific labeled immunoconjugates of interest for nuclear medicine. Enzymes used in this field, including microbial transglutaminase, sortase, galactosyltransferase, and lipoic acid ligase, will be overviewed and their recent applications in the radiopharmaceutical field will be described. Since nuclear medicine can benefit greatly from the production of homogenous derivatives, we hope that this review will aid the use of enzymes for the development of better radio-conjugates for diagnostic and therapeutic purposes.

## 1. Introduction

Bioconjugation techniques that exploit enzymes are a growing field of protein chemistry due to the interest to produce conjugates of proteins with small molecules or macromolecules for applications in research as well as in the biotechnological and pharmaceutical industries [1]. Traditional protein conjugation techniques rely mainly on the chemical derivatization of Lys residues with the drawback that since several Lys are present in a single protein, the reaction product is heterogeneous in respect to the site of conjugation. Hence, if the protein conjugate is to be used for pharmaceutical applications, it is of primary importance to characterize the different positional isomers for their respective chemical, biological, and pharmacological properties. The pharmaceutical industry is thus particularly interested in methods that allow for the site-specific modification of proteins because they can reduce the analytical efforts to characterize the reaction product and lead to a better performing protein-based drug.

Proteins have shown unique potential to improve human health both as therapeutics and as drug vectors. As therapeutics, the diversity and biospecificity in protein function impart the ability to treat different pathologies including cancer, autoimmune, metabolic, and infectious diseases [2]. In addition, proteins are also ideal scaffolds to deliver payloads to a specific biomarker. In the field of cancer treatment, numerous monoclonal antibodies (mAb) have been developed as protein-based therapeutics that specifically accumulate at the tumor site and activate the neutralization of malignant cells. The ability of mAb to successfully target the malignant tumor has been also exploited for imaging and therapeutic purposes by combining these proteins with imaging probes such as contrast and optical agents, pertinently selected diagnostic or therapeutic medical radionuclides, and small cytotoxins to increase their effect on tumor cells. Among these, radiolabeled proteins are very promising to cover an important role in the development and implementation of personalized targeted treatment of cancer and its metastasis [3,4,5]. Actually, nuclear molecular imaging (MI) with radiolabeled proteins based on therapeutic mAb or on different formats derived from immunoglobulin G (IgG) and from alternative scaffolds is utilized as a scouting procedure before radiotherapy. Indeed, it allows for confirming the tumor targeting and accurately estimating the radiation dose delivered to both tumor and healthy tissues, giving an important help for the selection of the candidates for radioimmunotheraphy (RIT). In this connection, while the development of new specific mAb and related is fundamental to target different tumors and more histotypes for the same tumor, the development of methods that allow for an efficient and site-specific derivatization of proteins in physiological conditions (<37 °C, pH 6–8, aqueous media) so as to not perturb protein structure and function, is equally important [6].

With this aim, different enzymatic approaches have been developed to produce homogenous immuno-conjugates that maintain the affinity for the antigen [2,3,4,5,6,7]. In this review, we focus on the recent developments in the use of enzymes for the production of radiolabeled antibodies and related studies. First, radionuclides and antibody formats used in nuclear MI and medicine are described (Section 3). Strategies currently used for the radiolabeling of biomolecules are overviewed in Section 4, with considerations on the limits of chemical methodology and the advantages of the enzymatic approaches in terms of chemoselectivity and site-specificity. A panel of four enzymes including microbial transglutaminase, sortase, galactosyltransferase, and lipoic acid ligase are discussed for their characteristics as tools for protein conjugation (Section 5) and for their recent applications in the radiopharmaceutical field (Section 6). Finally, the concluding remarks section aims to compare the different enzymes suggesting the pros and cons of their use in this specific field. Since radiolabeled antibodies and their derivatives are becoming instrumental for research studies and clinical management of different diseases, we hope that this review can provide a basis for further development of the use of enzyme conjugation in the production of better performing radioimmunoconjugates (RICs).

## 2. Introduction to Molecular Imaging

Molecular imaging is one of the most fast-developing areas of research. It aims to visualize, characterize, and quantify, in a non-invasive way, processes on molecular or cellular levels in living systems, giving clinicians important information both in the diagnosis and for monitoring the treatment of diseases [8]. Nuclear MI by single-photon emission computed tomography (SPECT) and, especially, by positron emission tomography (PET) provides unique advantages over classical diagnostic procedures, which mainly offer visualization of nonspecific changes related to morphology, enabling high detection sensibility, high-resolution images, and quantitative analysis of the tracer. Since the availability of a plethora of radionuclides (vide infra) with nuclear features suited for medical diagnosis and cancer therapy as well as for theranostic purposes, nuclear MI is characterized by remarkable flexibility in the design of radiolabeled probes and has shown the potential to speed up the diagnosis of diseases and the personalization of medical care.

Currently, molecular targeting is one of the most promising approaches to visualize and treat disseminated cancer. It is interesting to note that although the imaging tracers used in biomedical research and in clinical practice are generally based on small molecules and peptides, the research interest has shifted toward the development of radiolabeled naturally occurring or naturally inspired biomolecules, such as antibodies, especially IgG and their truncated counterparts (F(ab’)_2_, Fab, scFv) as well as engineered mAb fragments (miniboby, diaboby, nanobody), and small protein scaffolds (affibody, etc.) [3,9,10,11]. The utility of these biomolecules to treat cancer is due to their ability to bind tumor-associated antigens (e.g., HER2, EGFR, CD20, etc.) overexpressed on the surface of neoplastic cells and their paring with the unique advantages of radionuclides.

To date, looking at the radiopharmaceutical market, all RICs approved by the FDA and EMA are murine antibodies (Table 1). Moreover, most of them are diagnostic agents radiolabeled with gamma emitter radionuclides, ^111^In and ^99m^Tc, and the most recent approval occurred a decade ago [9]. In spite of this, the application of these agents in early phase clinical trials has increased dramatically in recent years [5,6], thanks to the continuous scientific and technological advances in the development of therapeutic mAb for the treatment of disease (as an example, 44 new mAbs gained approval by the FDA over the last five years, among which 10 were for cancer treatment) [2] and to the spread of mAb-based drug conjugates as new paradigm for the selective target delivery of drugs to disease tissues (mainly tumors and disseminate tumor cells) thus combining chemotherapy and immunotherapy [12,13].

**Table 1:** Radioimmunoconjugates that are approved by FDA and EMA.

Trade Name	Generic Name	Company	Approval Year		Antibody	Target	Cell Line	Radionuclide	Indications
			EMA	FDA					
**OncoScint**	Satumomab pendetide	Cytogen	NA	1992	B72.3, mouse IgG1	TAG-72	Hybridoma	^111^In	Colorectal and ovarian carcinoma
**CEA-Scan**	Arcitumomab	Immunomedis	1996 (withdrawn in 2005)	1996	IMMU-4, mouse IgG Fab’	CEA	Hybridoma	^99m^Tc	Colorectal cancer
**Myoscint**	Imciromab pentetate	Centocor	NA	1996 (discontinued)	R11D10, mouse IgG2a Fab’	Human cardiac myosin	Murine ascites	^111^In	Myocardial infarction
**Verluma**	Nofetumomab merpentan	Boehringer Ingelheim, NeoRx	NA	1996	NR-LU-10, mouse IgG2b Fab	carcinoma-associated antigen	Hybridoma	^99m^Tc	Breast, lung, gastrointestinal, ovary, bladder, kidney, cervix, and pancreas carcinomas
**ProstaScint**	Capromab pendetide	Cytogen	NA	1996	7E11-C5.3, mouse IgG1	PSMA,	Hybridoma	^111^In	Prostate carcinoma
**Zevalin**	Ibritumomab tiuxetan	Spectrum Pharms/Biogen	2004	2002	2B8, mouse IgG1	CD20	CHO	^90^Y	Non-Hodgkin lymphoma
**Bexxar**	Tositumomab	Corixa and GSK	NA	2002 (discontinued in 2014)	B1, mouse IgG2a	CD20	Hybridoma	^131^I	Non-Hodgkin lymphoma
**NeutroSpec (LeuTech)**	Fanolesomab	Palatin Technologies	NA	2004	RB5, mouse IgM	CD15	Hybridoma	^99m^Tc	Appendicitis
**Lymphoscan**	Bectumomab	Immunomedics		NA	LL2, mouse IgG2a Fab’	CD22		^99m^Tc	Non-Hodgkin lymphoma
**HumaSPECT**	Votumumab	KS Biomedix Ltd./Organon Teknika	1998 (withdrawn in 2003)	NA	88BV59, human IgG3	Cytokeratin tumor associated antigen	Human lymphoblastoid cell line transformed with EBV	^99m^Tc	Carcinoma of the colon and rectum
**Indimacis-125**	Igovomab	CIS Bio International	1996 (discontinued)	NA	OC125, mouse IgG1 F(ab’)2	CA-125		^111^In	Ovarian cancer
**LeukoScan**	Sulesomab	Immunomedics	1997	NA	IMMU MN3, mouse IgG Fab’	NCA-90 NS0		^99m^Tc	Osteomyelitis and appendicitis, including patients with diabetic foot ulcers
**Scintimun**	Besilesomab	CIS Bio	2010	NA	Murine IgG1	NCA-95	Hybridoma	^99m^Tc	Inflammation/infection

Antibody drug conjugates (ADC) and related can quickly be adapted for nuclear imaging through the conjugation of a pertinent radionuclide. This occurrence has driven the idea that radioimmuno-imaging can support in the understanding of therapeutic drugs both during preclinical studies and early phase clinical trials, providing unique information of the mechanism of action and failure of immunotherapy and guiding the rational for new drug development. However, beyond this, imaging probes are important tools in nuclear medicine oncology allowing one to: (a) evaluate the biodistribution of the therapeutic agent; (b) enable better patient selection and stratification to improve trial design; (c) confirm target expression and accessibility before the start of therapy, therefore patients who overexpress target receptors in disease tissues can be identified and a proper therapy defined. This permits to minimize the number of patients who might fail to benefit from therapy and to monitor the effectiveness of the treatment, providing real-time data on early clinical response; (d) assess the organs potentially at risk (i.e., side effects or off-target distribution); (e) determine the efficacy of treatment by measuring the accumulation of therapeutic drug into the tumor. As a result, one of the primary purposes in the creation of antibody-based radiotracers is not preparing stand-alone diagnostic probes but rather generating companion imaging agents that can guide the development and application of therapeutics [2].

## 3. Radionuclide and Antibody-Based Tumor Targeting Molecules for Radioimmunoimaging and Therapy 

The radionuclides used in conjunction with antibodies along with their nuclear properties, suitable chelating systems, and possible applications in PET/SPECT imaging and radioimmunotherapy (RIT) are summarized in Table 2. They can be classified according to their physical half-life (t_½_) as short (e.g., ^18^F, ^68^Ga, ^99m^Tc), intermediate (^64^Cu, ^86^Y), and long (^89^Zr, ^124^I, ^111^In) living radionuclides. The availability of a wide range of radiometal ions makes possible to careful pick the specific nuclear properties that are needed for a vast number of different applications [14,15,16]. In contrast to standard PET radionuclides, radiometals offer several advantages, including a wide range of half-lives to better match the biological process of interest, and relatively mild labeling conditions facilitating their use with sensitive biomolecules, such as antibodies [15,16]. 

When selecting the radionuclide to tag a mAb or its truncated derivatives, the most important factor to take into account is that the radiation dose to the tumor is to be optimal when compared to that of healthy tissues, to reach a high target-to-non-target ratio. The most critical item to consider when choosing a radionuclide is matching the physical half-life of the radionuclide with the biological half-life of the biomolecules to provide synchronous activity. This is essential to ensure that there is sufficient time for the RIC to accumulate in the tumor site before the radionuclide decay, to allow for good tumor visualization, and that the radiation exposure to normal tissues is as low as possible [17]. Biomolecules used for nuclear immuno-imaging and therapy have different formats that define and impact on their pharmacokinetics and development (Table 3). The biological half-life of immunoglobulins and related molecules is mainly determined by the molecular weight and structure of the biomolecule. As a general rule, large proteins, such as intact mAb with molecular weight higher than 110 kDa, have long in vivo half-lives (weeks/days) that are responsible for low target-to-non-target ratios, and are cleared via the liver (Table 3). They are tagged with long-living radionuclides such as ^89^Zr and ^177^Lu, whereas biomolecules with molecular weight below this value have a biological half-life of hours and are cleared very rapidly via the kidneys. These fragments achieve optimal tumor-to-non-tumor ratios at ≥6 h after injection, allowing the attainment of good contrast and sensitivity on the day of injection or the day after injection, differently from intact mAbs that achieve this ratio typically at 4–7 days after injection. This consents the usage of medium-lived radionuclides as ^64^Cu and ^86^Y or relatively short-lived radionuclides as ^99m^Tc, with the benefit of an appreciable reduction of the adsorbed dose by patients compared with the use of ^89^Zr or ^177^Lu. However, radiolabeled (Fab’)_2_ and Fab are characterized by some issues attributable to their size. Both fragments are still too large for efficient extravasation and they are still above the enhanced permeability and retention (EPR) border. Moreover, Fab fragments are affected by a reduced binding affinity compared to IgG, due to the loss of the avidity effect of bivalent binding.

Single chain variable fragments, scFvs, are smaller than Fab fragments and have only one binding site like them, but scFvs are engineered to have sub-nanomolar affinity to the target. Nonetheless, although in vivo studies of radiolabeled scFvs display good imaging contrast, they may have quite low target uptake (2–4%IA), probably due to a suboptimal relation between blood clearance and extravasation rates. Engineered antibody fragments such as diaboby (dimeric bivalent form of scFv, ~50 kDa) or miniboby (fusion of scFv to the Fc region of IgG, ~80 kDa) allows for higher target uptake (in the range of 20–32%IA) but with the cons of high unspecific uptake and off target accumulation. Such constructs permit good imaging contrast within 24 h post injection. 

Single-domain antibody fragments, also known as nanobodies (sdAb or VHH), are isolated from immunized camelids [18]. They are the smallest fragments capable of specific binding to antigens by detecting specific disease markers in the fields of oncology, inflammation, atherosclerosis. Thanks to their good stability and low dimension, they are characterized by efficient extravasation, rapid blood clearance, and uniform tumor distribution with minimal unspecific accumulation. Good imaging contrast is achieved as early as 1 h after administration. Rapid clearance of sdAb permits labeling with short-lived radionuclides as ^99m^Tc, ^18^F and ^68^Ga. A common problem for these biomolecules is the high renal reabsorption, a feature common for many other small proteins including small peptides.

Among small protein scaffolds with sufficient affinity for in vivo molecular imaging, affibodies are very stable and highly water-soluble α-helical proteins with facilitated conjugation chemistry [19]. These biomolecules are small (58 amino acids) engineered proteins that can be selected to bind with a nanomolar affinity a large variety of cancer-associated molecular targets including HER2, EGFR, VEGFR2, PD-L1, etc. [19]. The most important feature of this class of molecules is the rapid refolding in the physiological milieu after denaturation, which permits the use of high temperature (95 °C), pH over the range 3.5–11.5, and organic solvents during the labeling and purification processes. In particular, thanks to their small size, resulting in rapid blood clearance, good tumor penetration, and high binding affinity to selected targets, affibodies are considered ideal candidates for imaging purposes [19]. Additional advantages include the amenability to the site-specific incorporation of a variety of chelators and prosthetic groups suitable for radiolabeling in any desirable position by peptide synthesis methods as well as the introduction of a single Cys residue to permit site-specific modification by mean of maleimido-mediated thiol-directed chemistry [10,19].

**Table 2:** Radionuclides used in radioimmunoconjugates for PET, SPECT, and radioimmunotherapy *(RIT)*.

Radionuclide	Decay					Common Production Process ^a^	Chelator ^c^ Labeling Conditions	Properties
	t_½_ (h)	β^+^_max_ in KeV (Yield)	β^−^_max_ in keV (Yield)	γ in keV (Yield)	α in keV (Yield)			
**Alogens**								
**^18^F**	1.83 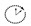	634 (97%)	140 (41%)			Cyclotron: ^18^O(p,n)^18^F (radionuclide/tracers can be transported over short distances)	^18^F-labeled prosthetic groups containing click chemistry handles, e.g., azides, [^18^F]FEA; or alkynes [^18^F]-FB-DBCO for SPAAC reactions; TCO—and tetrazine for IEDDA reactions	Only suitable for imaging of fast-clearing antibody fragments by PET; Cons: imaging up to 6 h after injection. Defluorination can occur resulting in bone-seeking radionuclide
**^123^I**	13.2 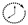	_	_	160 (83%)		Cyclotron ^123^Te(p,n)^123^I		Suitable for imaging of non-internalizing antibody fragments by SPECT; cons: dehalogenation can occur resulting in thyroid uptake
**^124^I**	100.2 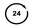	2138 (24%)	_	0.6 (61%)		Cyclotron: ^124^Te(p,n)^124^I (transportation worldwide including RICs)		Ideal for IgG imaging by PET with non-internalizing mAbs; cons: dehalogenation can occur resulting in thyroid uptake
**^131^I**	8.03 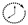	_	0.63 (90%)	0.36 (82%)		Nuclear reactor ^130^Te(n,γ)^131m,g^T → ^131^I (transportation worldwide)		Used for RIT; cons: dehalogenation can occur resulting in thyroid uptake.
**Metals**								
**^44^Sc**	3.9 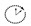	1474 (94%)		1157 (6%)		Sc(p, 2n) ^44^Ti → ^44^Sc (Generator)	DOTA 95 °C, 20–30 min, pH 4.0. Lower temperature need to extension of incubation time (hours)	Ideal for RIT with intact IgG and small scaffold proteins. Genuine theranostic.
**^47^Sc**	80.4 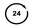		162	159 (68.3)		Nuclear reactor ^47^Ti(n,p)^47^Sc ^46^Ca(n,γ)^47^Ca → ^47^Sc		
**^64^Cu**	12.7 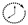	653 (18%)	579 (39%)			Cyclotron: ^64^Ni(p,n)^64^Cu (tracers can be transported over short distances)	NOTA/NOTA-type: fast complexation at RT (30–60 min; pH = 5.5–6.5); high kinetic inertness in vivo. Sarcophagine-type Diamsar: quantitative radiolabeling at RT in 2–30 min; pH = 2–9 by using 10^−6^ M of chelator; compounds have excellent in vivo stability.	Relatively short t_½_ for imaging antibodies, preferably suitable for imaging of small antibody fragments by PET. Genuine theranostic.
**^67^Cu**	61.8 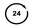		576 (20%), 482 (22%), 391 (57%)	184 (49%)		High energy cyclotron: ^68^Zn(p,2p)^67^Cu (not easily available)		Suitable for IgG imaging small antibody fragments by SPECT and RIT. Genuine theranostic.
**^67^Ga**	78.3 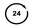			93 (39%), 184 (21%), 300 (17%)		Cyclotron ^68^Zn(p,2n)^67^Ga or ^67^Zn(p,n)^67^Ga(transportation worldwide)	DOTA: 37 °C, >30 min, pH 4.0–5.5 No optimal	Ideal for imaging with intact IgG by SPECT.
**^68^Ga**	1.13 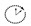	1899, 822 (90%)		108 (3%)		^nat^Ga(p,xn)^68^Ge → ^68^Ga (Generator)	NOTA: RT, 30–60 min, pH 4.0–5.5. Stable	Only suitable for PET-imaging of fast-clearing antibody fragments.
**^86^Y**	14.7 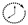	3141 (34%)		1.0(83%)		Cyclotron ^86^Sr(p,n)^86^Y	DOTA: 25–100 °C, 15–90 min, pH 4.0–6.0. Stable. NOTA: RT, 5 min, pH 4.0.Stable	Relatively short t_½_for imaging antibodies, only suitable for imaging with small antibody fragments by PET. Forms an ideal theranostics pair with ^90^Y.
**^90^Y**	64.1 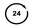	_	2280 (100%)			^235^U(n,f)^90^Sr → ^90^Y (Generator)nuclear reactor: ^90^Zr(n,p)^90^Y		Only RIT; forms an ideal theranostics pair with ^86^Y, ^90^Sr.
**^89^Zr**	78.4 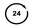	902 (23%)	_	0.9 (99%)		Cyclotron: ^89^Y(p,n)^89^Zr (transportation worldwide including RICs)	DFO: 25 °C, 60 min, pH 7–7.3.	Ideal for IgG imaging by PET, also with internalizing mAb; Cons: residualization in organ of mAb catabolism (liver, spleen, kidneys); demetalation, bone-seeking radionuclide.
**^99m^Tc**	6.02 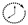	_	_	142 (89%)		^235^U(n,f)^99^Mo→^99m^Tc (Generator)	N_3_S- RT, pH 7 >60 min HYNIC- RT, pH 7 > 60 min. Tc(CO)_3_^+^- His-Tag RT, pH 7 > 60 min	Only suitable for imaging of fast-clearing antibody fragments by SPECT; Pros: cheap and easily available.
**^111^In**	67.3 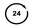	_	_	172, 245 (100%)		Cyclotron ^112^Cd(p,2n)^111^In^111^Cd(p,n)^111^In	DOTA: 37–100 °C, 15–60 min, pH 4.0–6.0. Stable	Ideal t_½_ for IgG imaging by SPECT, Cons: bone-seeking radionuclide
**^177^Lu**	159.5 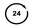	_	177 (12%), 385 (9%), 498 (79%)	112, 208 (100%)		Nuclear reactor ^176^Lu(n,γ)^177^Lu	DOTA: 25–100 °C, 15–90 min, pH 4.0–6.0. Stable NOTA: RT, 30–60 min, pH 4.5. Stable	RIT and imaging (SPECT) possible at the same time Genuine theranostic
**^225^Ac**	240 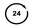				5600–5830 (100%)	^226^Ra(p,2n)^225^Ac ^232^Th(n)-^233^U-^229^Th-^225^Ac (Generator)^b^	DOTA: 37–60 °C, 30–120 min, pH 6.0.	RIT
**^213^Bi**	0.76 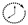		5869 (97.8%)		5549 (2.2%)	^227^Ac(n,γ)^229^Th ^228^Th(n,γ)^229^Th-^225^Ac-^213^Bi(Generator) ^b^	DOTA: 95–100 °C, 5 min, pH 6.0–8.7 no suitable for proteins. 3p-C-DEPA: RT, 5–10 min, pH 5.5 NOTA: RT, 5 min, pH 4.0. Stable	RIT
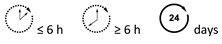								

^a^ [20]; ^b^ [21]; ^c^ [22,23] **EA**, ethyl azide; **DBCO**, Dibenzocyclooctyne; **SPAAC**, strain-promoted [3 + 2] azide-alkyne cycloaddition reactions **TCO**, *trans*-cyclooctene; IEDDA, inverse electron-demand Diels-Alder reaction. [14] **DOTA**, 1,4,7,10-tetraazacyclododecane-1,4,7,10-tetraacetic acid; **Diamsar**/**SarAr** 1-*N*-(4-Aminobenzyl)-3,6,10,13,16,19-hexaazabicyclo [6.6.6]-eicosane-1,8-diamine (SarAr); **NOTA**, 1,4,7-triazacyclononane-1,4,7-triacetic acid; **DFO**, Desferrioxamine B; **3p-C-DEPA**, 2-[(carboxymethyl)]-[5-(4-nitrophenyl-1-[4,7,10-tris-(carboxymethyl)-1,4,7,10-tetraazacyclododecan-1-yl]pentan-2-yl)-amino]acetic acid. **N_3_S**, mercaptoacetyltriglycine; **HYNIC**, 6-hydrazinopyridine-3-carboxylic acid, Tc(CO)_3_^+^-His-Tag.

**Table 3:** Summary of the key properties of intact antibodies, antibody fragments, nanobodies, and affibodies [10,17].

	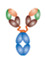	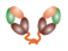	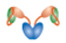	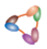	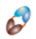	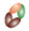	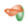	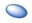	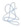
	IgG	F(ab’)2	Minibody	Triabody	Diabody	Fab	scFv	Nanobody	Affibody
**MW (kDa)**	~150	~110	~75	~75	~50	~50	~25	~15–12	6
**Avidity**	bivalent	bivalent	bivalent		bivalent	monovalent	monovalent	monovalent	monovalent
**Target specificity**	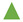	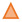	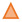	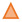	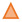	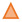	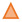	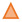	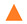 / 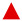
**Tumor uptake**	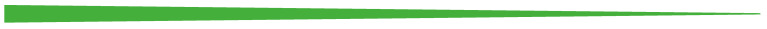								
**Tumor penetration**	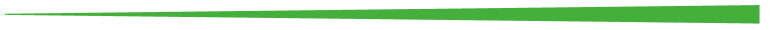								
**Clearance rate**	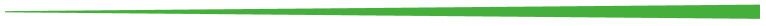								
**Excretion route**	Hepatic	Hepatic/renal	Hepatic	Hepatic	Renal	Renal	Renal	Renal	Renal
**Blood t_1/2_**	1–3 w	1–7 d	5–10 h		3–5 h	12–20 h	2–4 h	30–60 min	30–60 min
**Isotope t_1/2_**	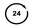	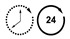	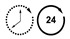	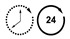	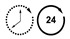	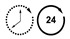	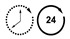	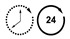	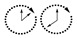
**Target/non-target**	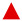	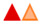	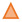	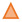	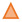	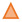	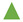	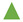	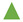
**Imaging (p.i)**	4–7 d		1 d	1 d	1 d	1 d	<1 d	<1 d	<1 d
**Radiolabeling process complexity**	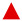	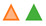	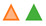	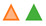	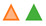	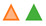	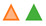	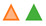	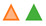
**Complexity development & test**	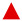	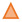	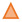	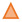	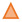	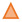	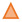	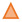	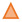
**Approval process & complexity of clinical translation**	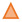	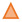	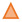	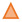	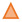	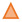	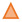	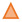	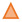
w = weeks; d = days; h = hours			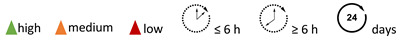						

## 4. Overview of Radiolabeling Strategies for Biomolecules

**Figure 1:**
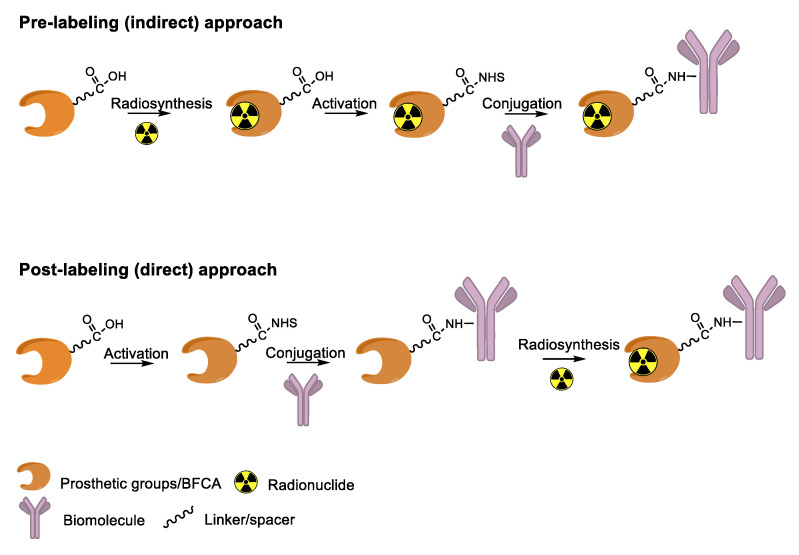
Radiolabeling approaches.

Antibody derivatization is the major challenge for the design and production of efficient RICs to preserve the affinity of the antigen-binding site for the target. With the exclusion of some limited examples of direct radiolabeling protocols (e.g., the radioiodination of tyrosine residues or the coordination of ^99m^Tc to thiolate groups of Cys side chains), the incorporation of radionuclides into mAb and relates is usually attained by using (i) a pre-labeling (indirect) approach for which a reagent is first radiolabeled and then conjugated to the biomolecule or (ii) a post-labeling (direct) approach in which the biomolecule is functionalized with a pertinent group that allows for the successive radiolabeling (Figure 1). 

The pre-labeling method is commonly used to tag biomolecules with nonmetallic radionuclides (e.g., fluorine-18). This strategy makes use of prosthetic groups: small molecules comprising of two domains that allow for both radiolabeling and subsequent conjugation to the biomolecule. In such cases, the incompatibility of the biomolecule with radiolabeling conditions (e.g., high temperature, non-physiological pH) is often the rationale behind the adoption of the indirect approach.

The post-labeling method is regularly employed for the tagging of biomolecules with radiometals via the so-called bifunctional chelating agents (BFCA). To date, this is the most used strategy, and it is particularly suited for the development of radiopharmaceutical “instant cold kits”. Usually, BFCAs are low molecular weight molecules designed to toughly coordinate the metal radionuclide by a variable combination of N, O, and S donor atoms and to carry additional functional groups to form a strong covalent bond with the biomolecule (BFCA-biomolecule). A limitation of this strategy may be the attainment of radiolabeled constructs with low apparent molar activity. Indeed, because of the micro- to nanomolar range concentrations of radiometals present in the reaction mixtures, during radiolabeling, there is almost always a large excess of the BFCA-biomolecule (in the micromolar range). As a result, due to the close chemical analogy, radiolabeled and non-radiolabeled proteins are difficult to be separated by using common purification techniques. Once injected, the non-radiolabeled biomolecule can compete with the radiolabeled one for binding sites, lowering the uptake of the latter in the tissue of interest and compromising the result of the diagnostic investigation. Therefore, the optimization of the apparent molar activity (radioactivity/mol; GBq/µmol) of the radiolabeled construct is critical, and it can be achieved using a precursor-to-radiometal ratio as low as possible for the quantitative complexation of the radiometal.

Having understood that both functionalization and radiolabeling procedures must keep the biological properties of native biomolecules unaltered, whatever the approach, two other important aspects need to be considered to generate effective homogenous immuno-conjugates: the *chemoselectivity* and *site-specificity* of the bioconjugation reaction [24,25]. The first is referred to the ability of a reagent to react selectively with only one type of functional group in the presence of other potential reactive groups. Meanwhile, the second is related to the ability to modify a biomolecule at a single defined position (or, in some cases, in a small number of defined positions). 

Biomolecules have many different functional groups (carboxylic acid, amides, amines, alcohols, thiols) and multiple copies of each type of them located at different positions of the primary sequence. This makes both chemo-selectivity and site-specificity difficult tasks. The use of readily available chemical reagents or BFCAs for the direct or indirect radiolabeling of biomolecules often leads to poorly chemically defined biologics that are mixtures of conjugates with heterogeneity both in the BFCA-to-biomolecule ratio and in the site of conjugation. The excessive derivatization of the biomolecule with BFCA as well as the random conjugation to positions that may be critical for biological activity, can significantly modify the in vivo performance of the RIC in terms of pharmacokinetics, selectivity/affinity against the molecular target, and stability of the final product [26]. In addition, the lack of site-specificity and homogeneity can become a serious concern, especially in view of the clinical translation of the radiolabeled molecules. Thus, it has become clear that exerting precise control over bioconjugation is vital for the development of effective RICs. 

The chemical functionalization strategies are a standard method in radiopharmaceutical chemistry for the chemo-selective modification of mAb and relates. The majority of such conventional chemical strategies have been relied on the reaction between the Lys or Cys side chains with chemicals bearing activated carboxyl groups (benzyl isothiocyanates or and N-hydroxysuccinimidyl esters) or thiol specific reagents (maleimide-containing derivative), respectively. Despite of both these methods are efficient and provide quite stable bioconjugates by forming amide or thioether linkages, they lack specificity, in particular in the case of Lys. Moreover, it was found that, in some circumstances, reactions involving sulfhydryl groups are reversible in vivo, resulting in the release of the maleimide group in plasma [27].

Click reactions are other widely used and well-characterized methods for efficient and selective protein derivatization. They are based on bioothogonal functional groups, which promptly react with moieties not typically present in biological systems, thus enabling the chemoselective and site-specific derivatization of a protein. Although copper-catalyzed azide–alkyne reaction (CuAAC) has been shown to be efficient and selective, there are some important limitations that reduce its usage both in immunoconjugates preparations as well as in radiopharmaceutical applications [24]. The presence of copper ions can damage the protein structure and interfere with their function. CuAAC cannot be used in combination with radiometal chelators because the presence of micromolar amounts of Cu catalyst can interfere with the chelation chemistry of radiometals, often present in nanomolar concentrations. The recent introduction of strain-promoted azide–alkyne cycloaddition (SPAAC) reaction and, most recently, of the inverse electron-demand Diels–Alder (IEDDA) cycloaddition between an electron-rich dienophile (such as trans-cyclooctene, TCO) and an electron-deficient diene (e.g., tetrazine, Tz) has allowed for bypassing such restrictions. Cu-free click reactions actually have been successfully employed in a variety of applications related to radiosynthetic methodology; as an example, IEDDA ligation has been utilized to assist a modular strategy for the radiolabeling of antibodies with positron-emitting radiometals, and it has also demonstrated great utility for the radiolabeling of sensitive peptides and proteins with ^18^F-labeled prosthetic groups (vide infra) [6]. Either way, the use of bioorthogonal reactions requires the specific incorporation of bioorthogonal moieties into the protein backbone that has been achieved by exploiting Cys residues, or by using engineered proteins comprising azide- and alkyne-containing unnatural amino acids [24].

To overcome these limits, over the last decade, taking advantage from the substantial breakthrough in the field of bioconjugation techniques, conjugation based on enzymatic methodologies has gained attention and has been applied in the preparation of targeting constructs for nuclear MI as a valid alternative. Indeed, these strategies are more selective, allowing for site-specific and stoichiometric mAb modification, and they offer highly versatile chemistry in pseudo-physiological conditions, thus preserving the structure and function of the native biomolecules (vide supra) [1]. In this connection, the introduction of such useful Cu-free bioorthogonal alternatives has given a substantial impetus to the so-called chemo-enzymatic approaches (which combine chemical and enzymatic transformations) to yield homogeneous RICs and derivatives. These would not only extend the range of applicability of enzymatic methods but would also minimize the synthesis and purification steps, reducing the cost of the production process. The adequate refinement of the chemo-enzymatic strategies may also reduce time-consuming production steps and maximize the radiochemical yield that are especially needed for short-lived radionuclides such as ^18^F, ^68^Ga and ^99m^Tc. 

## 5. Enzymes Used for the Site-Specific Derivatization of Proteins

### 5.1. Microbial Transglutaminase 

**Figure 2:**
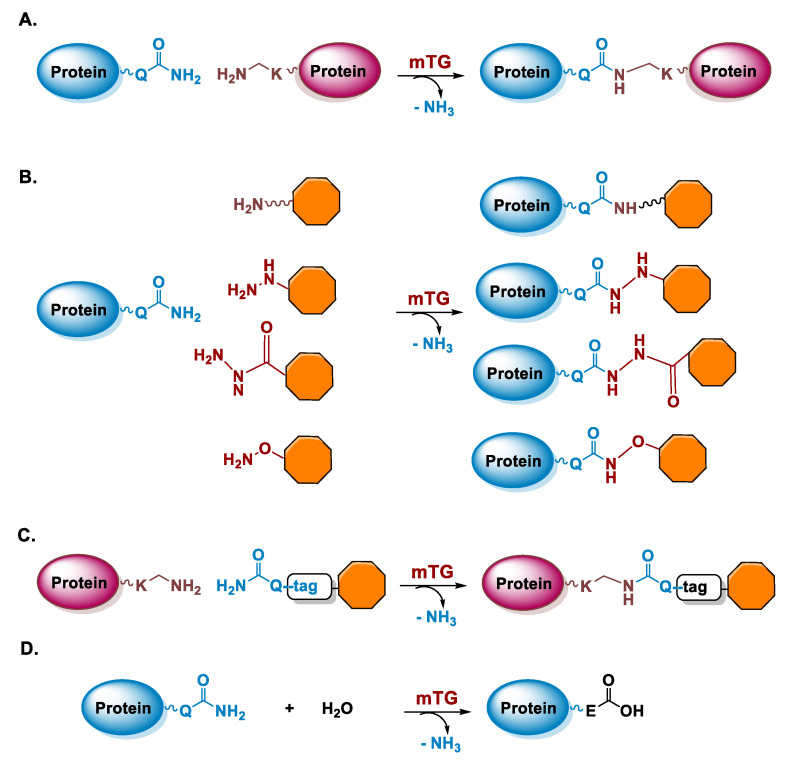
Reactions catalyzed by mTG. (**A**) If a Gln residue and the ε-amino group of a Lys residue of two proteins are involved, the reaction generates the formation of an isopeptide bond between the two residues leading to protein crosslinking. (**B**) If the primary ammine, hydrazine, hydrazide, or alkoxyamine belong to a ligand, the reaction leads to protein derivatization at the level of a Gln residue. (**C**) The ligand can also be functionalized with a Q-tag allowing protein conjugation at the level of Lys residues. (**D**) In the absence of primary ammines, in aqueous solution glutamine deamidation occurs, resulting in the conversion of glutamine into glutamic acid.

Transglutaminases (TGase; EC 2.3.2.13) are enzymes that catalyze the cross-linking between ε-amino group of Lys residues and γ-carboxamide group of Gln residues of proteins through the formation of ε-(γ-glutamyl)lysine isopeptide bonds, which are stable and protease resistant, and the release of ammonia (Figure 2A) [28]. TGases are a large family of enzymes detected in several organisms, including mammals, invertebrates, plants, and microorganisms [29]. In mammals, TGases are involved in important physiological functions, such as blood coagulation and keratogenesis and pathological processes as cancer and tissue fibrosis [30]. The TGase reaction is nowadays also widely exploited for the production of protein derivatives, in tissue engineering and in food and leather processing [31,32,33,34,35]. For industrial applications and protein conjugation, bacterial TGases are preferred which show little sequence similarity with mammalian TGases [29,36]. In particular, a TGase isolated from *Streptomyces mobaraensis* called microbial transglutaminase (mTG) offers several advantages in respect to mammalian TGases as calcium-independence, nearly half molecular mass, a lower substrate specificity, a lower deamidation activity, and availability in large quantities and with lower costs [37,38]. For the purposes of enzymatic-mediated radiolabeling, mTG is the enzyme used for the applications discussed in this review. In the following, we will give an overview of the characteristics of the mTG-catalyzed reaction. 

#### 5.1.1. Determinants for the Site-Specificity of the mTG-Catalyzed Reaction

Microbial TGase is a monomeric protein with a molecular mass of 37.9 kDa and a measured isoelectric point of pH 8.9 [37]. The 3D structure of mTG has been solved and it consists of a compact domain with a disk-like shape in which the catalytic triad is composed of not-contiguous Cys64, Asp255, and His274 residues and it is located at the bottom of a cleft with a depth of 16 Å [39]. The key steps for catalysis involve the interaction of the γ-carboxamide group of a Gln residue of a substrate with the TGase active site and its reaction with the Cys64 residue leading to the formation of a reactive thioacyl moiety. This thioester intermediate then reacts with an amino donor, thus leading to the formation of an isopeptide amide bond (Figure 2A–C). In the absence of a reactive Lys residue or primary amine, water leads to the hydrolysis of the acyl intermediate resulting in the deamidation of the Gln residue and its substitution with glutamic acid, (Figure 2D). The crystal structure of mTG is quite useful to understand the reactivity of the enzyme. Indeed, several acidic residues are located in the active site cleft explaining the low reactivity of the enzyme toward negatively charged substrates due to an unfavorable interaction. On the other end, hydrophobic compounds show a good affinity and actually patches of hydrophobic residues are located on the surface around the active site [39].

With the aim of protein derivatization, mTG can modify a protein if it contains reactive Gln or Lys residues. This possibility constitutes an advantage of mTG in respect to other enzymes used for protein conjugation, which generally require the introduction of a specific recognition sequence by recombinant methods [1]. Indeed, there is always the possibility that a change of the polypeptide sequence of a protein can affect its structure and activity and in the case of protein drugs it can increase the risk of immunogenicity. In the absence of a “natural” reactivity, through recombinant expression, a reactive peptide tag containing a Gln or Lys residue (Q-tag and K-tag, respectively) can be inserted in a specific position of the polypeptide chain or can be fused at the C-terminus or N-terminus of the protein [40,41]. It has been reported that also the N-terminus of a polypeptide chain starting with a peptidyl tag of three Gly residues can be conjugated to a Gln residue by mTG [42]. Importantly, in the case of mTG, tags are not necessarily inserted at the N- or C-termini, as it is required for most enzymes used in protein conjugation. Q- and K-tags can instead also be inserted within the sequence of the protein [41,43] or even single Gln or Lys residues can be introduced at internal locations of the sequence as demonstrated for the purposes of site-specific antibody conjugation [44,45]. These diverse possibilities to introduce reactive Gln and Lys residues allow flexibility in the design of an optimal protein mutant that can be derivatized by mTG. 

Regarding the derivatization of reactive Gln and Lys already present in the sequence of proteins, especially in the case of Gln residues, several cases of site-specific protein modification by mTG have been reported in which the sites of conjugation have been identified (Table 4) [46]. The selectivity of the reaction is quite impressive since among several Gln or Lys residues only a few are accessible for derivatization with high yield allowing the site-specific modification of the protein [47]. In different studies of protein conjugation by mTG, we found that in most cases reactive Gln and Lys residues are embedded in flexible regions of a protein structure [46,48,49,50,51,52]. To prove this correlation, we studied the reactivity of mTG to myoglobin (Mb), a protein that is a model of protein structure and folding and thus well characterized from a conformational point of view [48,53]. Mb is a small monomeric heme-containing protein of 153 amino acids, that in the holo-form shows a globin fold constituted by eight helices (named A–H). We observed that if we induce partial unfolding of this protein at neutral pH by removing heme, the derivatization exerted by mTG occurs selectively at the level of Gln91 or Lys96 and Lys 98 residues located in the region of helix F (residues 82–97), that is known to become disordered in the apo-form of Mb (Table 4) [54]. Moreover, in the case of antibody derivatization, the reactive Gln295 residue, which is located in the C/E loop (residues 295–299) of the CH2 domain in the heavy chain of IgG1 is modified by mTG only after deglycosylation of the nearby Asn297 residue (Table 4) [45,55]. Based on the crystal structures of glycosylated and deglycosylated Fc fragments, it has been proposed that removal of the glycan increases the flexibility of the loop region, which promotes the derivatization of Gln295, even if in this case also steric hindrance of the glycan chain could impede mTG derivatization. Similarly, an aglycosylated IgG1 can be selectively derivatized at Lys288/290 and Lys340 residues, that are all located on the CH2 domain of the heavy chain on flexible loops (Table 4) [56]. Flexibility at the level of the polypeptide chain is thus required for an effective interaction between the polypeptide chain containing the Lys and Gln residues and the Cys64 residue in the active site of mTG, as indicated also by other studies [57,58,59]. Analysis of the position of both Gln and Lys residues within the structure of a protein can be used as a criterion to predict potential sites of mTG derivatization.

**Table 4:** Examples of sites of mTG-mediated derivatization in proteins.

Protein (Organism)	N. AA	N. Gln/N. Derivatised Gln	Gln Sequences ^a^			N. Lys/N. Derivatised Lys	Lys Sequences ^a^			Ref.
			-5 -1		+1 +5		-5 -1		+1 +5	
Myoglobin, Mb (*Equus caballus*)	153	6/0 (holoMb) ^b^ 6/2 (apoMb)	LKPLA KELGF	**Q91** **Q152**	SHATK G----	19/0 (holoMb) ^b^ 19/2 (apoMb)	QSHAT HATKH	**K96** **K98**	HKIPI IPIKY	[48]
α-Lactalbumin, LA (*Bos taurus*)	123	6/0 (holoLA) ^c^ 6/4 (apoLA)	SGYDT TQAIV EYGLF WCKDD	**Q39** **Q43** **Q54** **Q65**	AIVQN NNDST INNKI NPHSS	12/1 (K122; holoLA) ^c^ 12/4 (apoLA)	ELKDL FQINN ALCSE QWLCE	**K16** **K58** **K114** **K122**	GYGGV IWCKD LDQWL L----	[48]
Avidin *(Gallus gallus*)	128	4/0	-			9/2	QNTIN RLRTQ	**K58** **K127**	RTQPT E----	[49]
Interferon α-2b (*Homo sapiens*)	165	12/1	EACVI	**Q101**	GVGVT	10/2	LFSCL ESLRS	**K31** **K164**	DRHDF E----	[50]
Interferon β-1a (*Homo sapiens*)	166	11/0	-			11/2	LEYCL DFTRG	**K33** **K115**	DRMNF LMSSL	[51]
Growth hormone (*Homo sapiens*)	191	13/2	YIPKE GQIFK	**Q40** **Q141**	KYSFL TYSKF	9/1	KQTYS	**K145**	FDTNS	[53,60]
Interleukin-2(*Homo sapiens*)	133	6/1	VLNLA	**Q74**	SKNFH	11/ND	-			[61]
Granulocyte colony-stimulating factor (*Homo sapiens*)	174	17/1	ALQPT	**Q134**	GAMPA	4/1	LCATY	**K40**	LCHPE	[53,62]
Granulocyte-macrophage colony-stimulating factor (*Homo sapiens*)	127	8/1	CWEPV	**Q126**	E----	6/0	-			[49]
Bacteriorhodopsin *(Halobacterium salinarum)*	249	4/1	---QA	**Q3**	ITGRP	7/1	VGALT	**K129**	VYSYR	[63]
IgG1 ^d^ (*Homo sapiens*)	HC: 451 LC: 213	HC:18/1 (degl.) LC: 12/0	KPREE	**Q295**	YDSTY	HC: 36/2 (agl.) LC: 13/0	EVHNA HNAKT TISKA	**K288** **K290** **K340**	TKPRE PREEQ ^e^ GQPRE	[45,56]
Notexin (*Notechis scutatus scutatus*)	119	3/0	-			11/6	KGCFP YCRNI CRNIK RNIKK WNIDT NIDTK	**K63** **K82** **K83** **K84** **K115** **K116**	MSAYD KKCLR KCLRF CLRFV KRCQ- RCQ--	[52]
G-actin (*Oryctolagus cuniculus*)	375	11/1	GRPRH	**Q41**	GVMVG	19/ND	-			[64]
Trypsin inhibitor, STI2 (*Streptomyces longisporus*)	110	1/ND	-			4/1	GVICN	**K70**	LYDPV	[65]
Dispase autolysis-inducing protein, DAIP (*Streptomyces mobaraensis*)	348	5/5	TTGTL HNDEL AGSDG YGTYF GLEEV	**Q39** **Q65** **Q144** **Q298** **Q345**	SVSYT RSTDA LYDST AYGTD IHH--	10/ND	-			[59]
Papain inhibitory protein, SPIp (*Streptomyces mobaraensis*)	110	3/1	DIPIG	**Q6**	KMTGK	6/ND	-			[66]
CRM_197_, mutant of diphtheria toxin (*Corynebacterium diphtheriae)*	535	16/ND	-			39/2	VDSIQ QKGIQ GIQKP	**K33** **K37** **K39**	GIQKP PKSGT SGTQG ^f^	[67]

^a^ Amino acids flanking reactive Gln and Lys residues. When more than one reactive residue is present, Gln and Lys residues evidenced in grey are the preferential sites of derivatization. ^b^ holoMb and apoMb refer to Mb with and without the heme group, respectively. ^c^ holoLA and apoLA refer to LA with and without calcium, respectively. ^d^ HC, heavy chain; LC, light chain; degl., deglycosylated; agl., aglycosylated. The number of residues of HC and LC and the number of Gln and Lys residues were calculated on the amino acid sequence of the antibody rituximab. ^e^ One site of derivatization is at the level of K288 or K290 (not distinguished). ^f^ One site of derivatization is at the level of K37 or K39 (not distinguished). AA = amino acids; ND = not determined.

In the absence of protein reactivity to mTG, Q- or K-tags can be inserted in the sequence of the protein and several different tags have been reported and recently reviewed [32,35]. From the sequences flanking reactive Gln and Lys residues in proteins, it cannot be derived a unique consensus sequence of amino acids to be used as mTG substrate (Table 4). However, the development of efficient peptide tags (Q-tag and K-tag) from the screening of combinatorial libraries of peptides or from the sequence nearby reactive Gln and Lys in proteins, has evidenced as amino acids at the C- and N-termini of Lys and Gln residues have an influence on their reactivity. In particular, a Pro residue at the C-terminus of Gln or Lys residues (e.g., Gln-Pro or Lys-Pro) slows down the derivatization mediated by mTG, while at the N-terminus it does not affect the reactivity [44,48,68,69]. Most studies on sequence preferences of mTG were performed on the reactivity of Gln residues. They showed a preference for hydrophobic residues (aromatic or aliphatic) adjacent to the Gln residue, while the presence of negatively charged amino acids inhibits the derivatization [69,70,71]. These results can be explained in light of the distribution of acidic and aromatic residues nearby the active site of the enzyme (vide supra) [39]. In general, different efficient Q-tags and K-tags are currently used, engineered into proteins by recombinant methods or linked to small ligands [40,72]. However, still there is not a unique consensus sequence for one highly reactive tag to be used for mTG derivatization [73]. 

For the purposes of protein conjugation, the substrate used for Gln modification has to contain a primary amine without steric hindrance and separated by a spacer of at least four carbon atoms from the payload (e.g., a fluorophore, a chelating agent), especially if this is negatively charged (Figure 2B) [74]. Indeed, even if mTG reacts with acyl acceptor substrates with very different chemical structures, ligands containing aromatic moieties show a higher reactivity while the presence of negatively charged groups is detrimental [75]. Recently, it has been demonstrated that mTG can catalyze the reaction also with non-canonical acyl acceptor substrates containing hydrazines, hydrazides, and alkoxyamines in place of the primary ammine (Figure 2B) [76]. These substrates lead to the formation of analogous of the isopeptide bond and they also allow the introduction of reactive groups into the sidechain of Gln residues for subsequent conjugation reactions. Instead, for Lys derivatization, a ligand containing a Q-tag is always required, such as the dipeptide carbobenzoxy-L-glutaminyl-glycine (ZQG) moiety or a longer peptide tag [32,35,37,70].

#### 5.1.2. Conditions of the mTG Reaction

The main advantage of the TGase-mediated protein conjugation compared to chemical reactions resides in the fact that the modification of a protein substrate occurs under physiological conditions, such as pH 6.0–7.0 at 37 °C [37]. The high selectivity of the reaction at the level of specific Gln or Lys residues leads to the production of homogeneous protein bio-conjugates that can be easily characterized in terms of site of derivatization as well as of biological properties. In the case of proteins that display both reactive Gln and Lys residues, there can be the production of dimers or oligomers of the protein by inter-chain crosslinking as a side product, but their yield can be lowered in the presence of an excess of ligand [50]. A percent of organic solvents or denaturing agents are also tolerated by mTG, and they can be useful to increase the selectivity of the derivatization [77,78]. For example, if the mTG reaction is performed in 50% (*v*/*v*) ethanol or 60% (*v*/*v*) methanol, human growth hormone is derivatized only at Gln141 level, while in the absence of solvent addition also Gln40 is derivatized (Table 4) [53,77]. Immobilization of mTG can also be beneficial to increase the site-specificity of the enzyme to obtain highly homogenous derivatives [56]. Indeed, avidin can be derivatized by mTG in solution at Lys127 and with lower yield at Lys58, leading to the formation of a lower percentage of a double derivative (i.e., 30%) (Table 4). When the same reaction is performed with immobilized mTG, avidin is modified almost selectively at Lys127, the double derivative being detected with a 2% yield. Clearly, immobilization of the enzyme allows to also avoid a further step of purification to remove mTG when the reaction has gone to completeness. Finally, a change in specificity of the modification has been obtained also by engineering mTG. A recent study reported the development of variants of mTG that can efficiently modify Gln295 in the heavy chain of IgG even in the presence of natural glycosylation [79].

### 5.2. Sortase 

**Figure 3:**
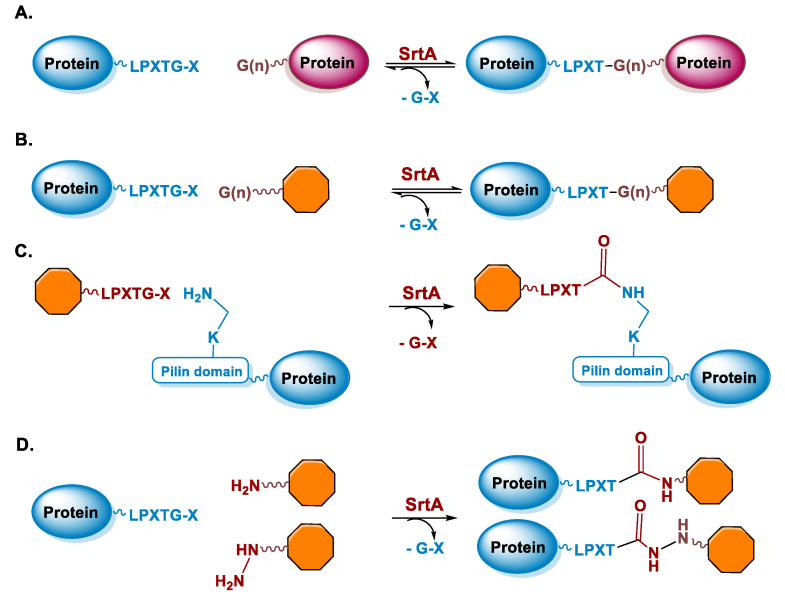
Reactions catalyzed by SrtA. (**A**) SrtA can catalyze the conjugation of a protein carrying the sortag sequence to another protein that has at the N-terminus an oligoglycine sequence. (**B**) The protein fused to sortag can be conjugated by SrtA to a cargo linked to an oligoglycine peptide. (**C**) The protein can also be fused to the pilin domain and it can be derivatized by SrtA with a ligand containing the sortag sequence. (**D**) Ligands containing primary amine or hydrazide groups can also be used.

Sortases are a family of enzymes widely present in Gram positive bacteria where they are functional to attach proteins to the cell wall and to assemble pili [80,81]. One of the most studied enzymes is Sortase A (SrtA) from *Staphylococcus aureus*, which has been developed as a tool for protein conjugation and semisynthesis (for a review see [82,83,84,85]). SrtA is a Ca^2+^ dependent Cys transpeptidase and in the bacterium it catalyzes the covalent binding of proteins at the level of lipid II, a peptidoglycan precursor [86]. The transpeptidase reaction catalyzed by SrtA involves surface proteins of the bacterium containing the sequence LPXTG (where X is any amino acid) in the C-terminal region and lipid II having an N-terminal pentaglycine (Gly)_5_ sequence. The enzyme active site displays a Cys residue that catalyzes the cleavage of the LPXTG motif between the Thr and Gly residues, leading to the formation of a protein-StrA thioacyl intermediate. The N-terminal amino group of the pentaglycine sequence then attacks the acyl-intermediate resulting in the formation of a new peptide bond (Figure 3A) [87]. StrA is formally classified as a Cys endopeptidase (EC 3.4.22.70) and indeed, in the absence of the (Gly)_5_ peptide, it hydrolyses the peptide bond between Thr and Gly. However, transpeptidation occurs faster than hydrolysis and in the presence of the (Gly)_5_ peptide it is favored [88]. This reaction is exploited for protein conjugation in which the sortase-mediated ligation allows for the site-specific conjugation between synthetic peptides or different chemical compounds and recombinant proteins through the formation of a peptide bond (Figure 3A,B) [89]. Indeed, the sequence tag LPXTG (named sortag) and an oligoglycine-terminating peptide (Gly)_n_ can be fused at the C-terminus and N-terminus of two proteins, respectively for protein crosslinking or one of the two can be conjugated to a ligand, allowing protein modification with different chemical moieties (Figure 3A,B).

The LPXTG sequence can be attached to the C-terminus of a protein, but it has to be followed by at least one additional residue, preferentially a Gly residue [90,91]. Alternatively, the sortag motif can be inserted within flexible regions of proteins [92]. On the side of the acyl acceptor substrate, the oligo-Gly tag can be reduced to a single Gly or substituted with the primary amine from Lys residues. This relaxed specificity towards the nucleophile has been exploited to derivatize a Lys residue present in a pilin domain peptide (Figure 3C) [93]. One or more pilin domain peptide sequences can be inserted at the terminal or internal sites of the target protein, allowing site-specific protein labeling with multiple copies of a small compound. The oligo-glycine tag can also be substituted with a primary amine or hydrazine and its derivatives (Figure 3D) [94,95,96]. The use of a poly-Gly nucleophile suffers from the drawback of the reversibility of the sortase-mediated reaction, which decreases the reaction yield. Indeed, after ligation, SrtA can cleave the resulting Thr–Gly peptide bond present in the product, while the released fragment (GX, Figure 3A,B) still has a glycine residue at its N-terminus that can function as an acyl acceptor. To prevent the reversibility of the reaction, alternative substrates such as those containing a primary ammine or hydrazine group can be useful since upon derivatization Gly is not present at the C-terminus of Thr impeding the StrA-mediated hydrolysis of the conjugate (Figure 3D). Other strategies are available to overcome the reversibility of the reaction [97], for example, increasing of the concentration of the reagents or enzyme immobilization that allows the easy product removal from the reaction mixture [98].

A limit of the SrtA reaction over the hydrolysis of the product is also the low turnover rate of the reaction that necessitates a long incubation time and high enzyme concentration. Further engineering of the enzyme has improved the reaction kinetics of StrA, and it has provided an enzyme that does not depend on the presence of calcium ions and that displays a higher stability to temperature and in the presence of organic solvents (for a recent review on StrA engineering see Freund and Schwarzer [99]). An interesting possibility of StrA derivatization is the co-expression in *E. coli* of StrA and of the target protein fused at the C-terminus to the sortag. This approach permits the direct purification of the modified protein from the cell lysate [100,101]. Indeed, if amine containing compounds can permeate the *E. coli* cells, then conjugation can occur in the cell after expression of the target protein and of the StrA enzyme.

### 5.3. Galactosyltransferase

**Figure 4:**
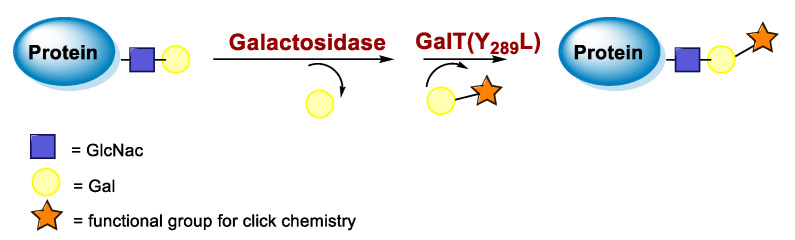
Protein derivatization with GalT(Y289L). A protein containing a carbohydrate chain with a terminal GlcNAc-Gal sequence (blue square and yellow circle for GlcNAc and Gal, respectively) is treated with a β-galactosidase to remove the terminal Gal, followed by the incorporation of a modified Gal sugar carrying a chemical handle using a mutant of β-1,4–galactosyltransferase, GalT (Y289L).

Protein conjugation can be specifically achieved even at the level of glycans that are present in many therapeutic proteins, as mAbs [102]. In antibodies, the glycan chains are located far from the antigen binding sites and at the level of the CH2 domain of the heavy chains of the Fc region. In particular, IgG1 are N-glycosylated in each heavy chain at the conserved residue Asn297 with a biantennary oligosaccharide that is partially galactosylated. In the protocol of this procedure, the two enzymes are combined (Figure 4). The first enzyme is a β-galactosidase (EC 3.2.1.23) that is used to hydrolyze galactose from the termini of the carbohydrate chains leaving a terminal N-acetylglucosamine (GlcNAc). The second enzyme is a mutant of β-1,4–galactosyltransferase (GalT (Y289L); EC 2.4.1.38) that can introduce a modified galactose sugar carrying a chemical handle in place of the removed galactose at the level of terminal GlcNAc [103]. A click chemistry reaction is then used to conjugate the protein at the level of the carbohydrate chain to a particular functionality. This approach has been developed for the site-specific bioconjugation of antibodies to produce homogeneous and well-defined immunoconjugates modified with different functionalities including biotin, fluorophores, toxins as auristatin F, and BFCAs for radiolabeling purposes (see below) [103,104,105,106,107]. Glycoconjugation protocols have been developed also with other endoglycosidases that expose core GlcNAc, thus allowing their derivatization with GalT (Y289L) or by attaching the functional moiety to other monosaccharides such as fucose and sialic acid [105,108]. A clear advantage of linking a cargo to the carbohydrate chain of a protein is that it does not require the mutation of the primary sequence to introduce a specific amino acid or a tag sequence.

### 5.4. Lipoic Acid Ligase

**Figure 5:**
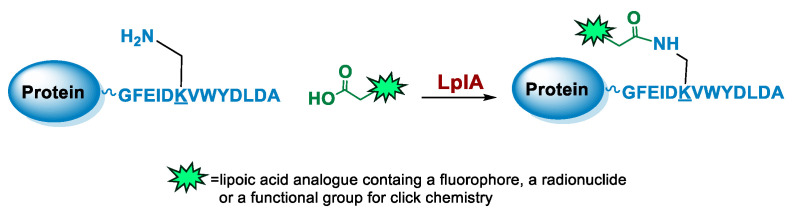
Protein derivatization with lipoic acid ligase. LplA derivatizes a protein fused to the 13 amino acid sequence of the LAP tag with a lipoic acid analogue. The enzyme catalyzes the formation of an amide bond between the ε-primary ammine of a Lys residue in the LAP tag and the carboxylate group of an alkyl carboxylate containing a functional handle.

Lipoic acid ligases (LplA) (EC 6.3.1.20) are enzymes that catalyze the derivatization of proteins with lipoic acid (lipoylation). The reaction is ATP and magnesium dependent and in *E. coli* LplA modifies specific Lys residues of proteins involved in oxidative metabolism [109]. Actually, LplA catalyzes the formation of a stable amide bond between the ε-primary ammine of a Lys and the carboxylate group of lipoic acid. The natural protein substrates were successfully reduced in size by in vitro evolution to a 13 residues sequence GFEIDKVWYDLDA named LAP (LplA Acceptor Peptide), that can be efficiently derivatized at the level of the Lys residue (Figure 5). Moreover, due to its small size, the LAP tag can also be easily fused to a target protein without interfering with its function [110]. Indeed, it can be attached to the protein at one of the two termini or at internal positions of the primary sequence localized in loop regions that do not affect protein structure and function [111,112]. The LAP tag thus enables a high site-specificity of derivatization but also some flexibility over the location of the modification, which is important since the optimal site of derivatization can be protein-dependent. Other important characteristics of the LplA-mediated ligation are the irreversibility of the reaction and the high derivatization rate that lead to high yields even at low protein concentrations [110]. This aspect is particularly important to avoid or simplify the purification of the protein conjugate [111,113]. 

LplA from *E. coli* has been established as a tool to obtain site-specific protein conjugation due to the ability of the enzyme to catalyze the derivatization of the recognized peptide tag with alkyl carboxylates containing a variety of functional handles [114,115,116]. A broader substrate specificity was obtained upon design of mutants of LplA at the level of residues of the lipoic acid binding pocket that allow the ligation of unnatural small molecules instead of lipoic acid. In particular, mutations of Trp37 to residues with smaller side chains enable to expand the substrate specificity of the enzyme [117]. If the size of the ligand cannot fit into the LplA active site, it can be introduced by a bioorthogonal ligation chemistry using the enzymatic attachment of reactive handles [112]. For example, Plaks et al. demonstrated that upon derivatization of the protein with an azide containing molecule at the level of the LAP tag, it is possible to efficiently chemically label the protein with poly(ethylene glycol) of 5 kDa, with a mannose moiety or with the fatty acid palmitic acid or to immobilize the protein [111]. In a recent paper, Wombacher and co-workers [118] tested a large panel of carboxylic acids containing dienophile and diene scaffolds for bioorthogonal cycloaddition reactions as substrates for the lipoic acid ligase mutant W37V. They selected the best performing substrates in terms of efficiency of ligation by LplA W37V and of performance in the cycloaddition reactions. This two-step chemo-enzymatic approach was then used to produce quantitative homogenous protein-protein conjugates. In particular, the therapeutic antibody trastuzumab fused to the LAP motif at the C-termini of the heavy chains was efficiently conjugated to EGFP and to the highly toxic monomethyl auristatin E. This approach holds promise for the production of antibodies conjugated to proteins or other cargoes and for other applications in which protein conjugation is needed.

## 6. Enzyme-Mediated Conjugation in Nuclear Molecular Imaging 

### 6.1. Transglutaminase-Mediated Conjugation in MI

In 2010, Schibli and co-workers were the first to use a method based on the TGase-mediated modification of antibodies for the site-specific incorporation of BFCAs, highlighting its great potential also in nuclear molecular imaging and radiotherapeutic applications [45]. In this groundbreaking study, authors exploited the features of mTG to prepare immuno-conjugates functionalized with different BFCA suitable for diagnostic and therapeutic radionuclides. Deferoxamine (DFO) and 4-(1,4,8,11-tetraazacyclotetradec-1-yl) methyl benzoic acid (CPTA) pertinently derivatized with cadaverine, were used as Lys-mimicking substrates of mTG to modify the anti-L1-CAM mAb, chCE7, and the commercial available anti CD20 antibody rituximab (RTX), both utilized in their deglycosylated form, since no modification of native mAbs catalyzed by mTG were achieved (Table 5, panel A) [119]. In this work, authors identified the Gln295 residue in the conserved glycosylation region of IgGs as the sole site for mTG-mediated conjugation, that becomes accessible only after deglycosylation by N-glycosidase F (PNGase F).

mTG-based conjugations were performed in mild conditions (phosphate buffer, pH 8.0; 37 °C) by incubating different mAbs (1 mg/mL) and substrates 400 µM with mTG (1 U/mL)) for 12–24 h depending on the mAb and substrate nature. Notably, when DFO was reacted with the mutant chCE7agl-antibody (agl = aglycosylated) in the presence of 1 U/mL of mTG, the reaction reached the plateau within 4 h. Moreover, in the enzymatic conjugation of DFO to deglycosylated chCE7 (chCE7degl), a twelvefold concentration of mTG was necessary (12 U/mL) to reach steady-state conditions within 4 h. Upon the conjugation reaction, a small number of BFCA was stoichiometrically and reproducibly conjugated to the proteins. Homogenous immunoconjugates with a BFCA-substrate/mAb ratio of exactly 2:1 were generated. Similar outcomes were also attained with the engineered chCE7agl mutant in which the two Asn297 residues were replaced by Gln to eliminate the N-glycosylation and thus, allowing the introduction of four chelators per antibody. Actually, completely homogeneous immunoconjugates with a substrate/mAb stoichiometry of 4:1 were attained (Hamblett showed that a drug/mAb ratio of 4 resulted in the optimal potential and safety of immunoconjugates [120]). Immunoconjugates were stably tagged (30 min at 37 °C) with different radiometals (^67^Ga, ^64/67^Cu, ^89^Zr) for in vitro and in vivo assessment of their pharmacological profiles. In vivo biodistribution studies were performed with engineered ^67^Ga-(DFO)_4_-chCE7_agl_ and ^64/67^Cu-CPTA-RTX in the pertinent animal model and compared with the biological profiles of the corresponding radio-immunoconjugates obtained by conventional chemical routes, for which only an average of BFCA/mAb ratio could be determined. Data showed for both the homogenous RICs superior distribution profiles with higher tumor-to-liver and tumor-to-kidney ratios when compared to those of immunoconjugates prepared by chemical coupling methods. Likewise, PET images of animals injected with enzymatically or chemically conjugated ^89^Zr-(DFO)_4_-chCE7_agl_ correlated well with the distribution data of ^67^Ga-(DFO)_4_-chCE7_agl_ analogues [45]. These findings are also valuable for simple drug immunoconjugates [121].

**Table 5:** Overview of TGase-mediated conjugation in radiopharmaceutical applications.

	Biomolecules	Bi-Functional Substrate	Radionuclide	Ref.
**A**	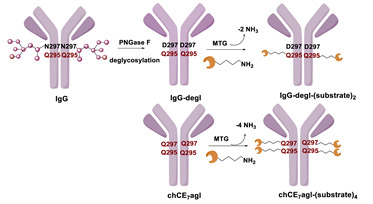	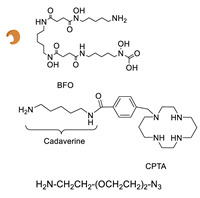	^67^Ga, ^64/67^Cu, ^89^Zr	[45]
**B**	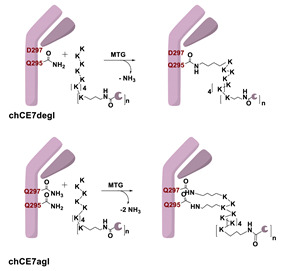	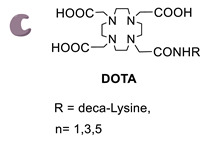	^177^Lu	[122]
**C**	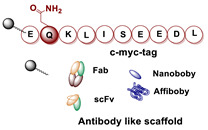	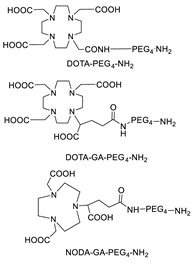	^111^In	[123]
**D**	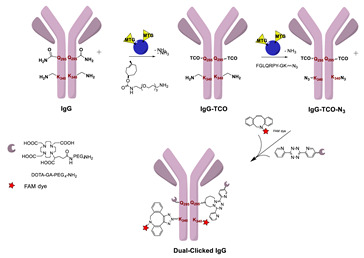	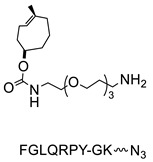		[56]
**E**	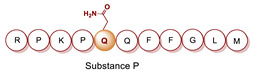	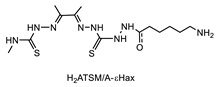	^99m^Tc; ^64^Cu	[124]
**F**	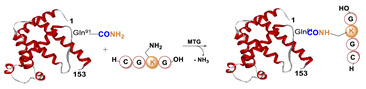	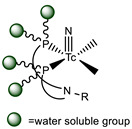	^99m^Tc	[125]

With the purpose of increasing the number of BFCAs specifically conjugated to the mAb and, consequently, of augmenting the substrate/mAb ratio as well as the specific activity of the RCI, in a subsequent work authors investigated the influence of different numbers of DOTA moieties (n = 1,3,5), coupled to a decalysine backbone, on the in vivo behavior of the chimeric monoclonal anti-L1CAM antibody chCE7 as chCE7degl and chCE7agl forms (Table 5, panel B) [122]. Decalysine backbone was selected to allow the recognition of the scaffolds by mTG. The reaction was performed in Tris-HCl buffer (0.04 M, pH 7.0) and potassium-free PBS buffer (pH 9.0) by incubating the mixture for 16 h at 37 °C. An mAb/ligand molar ratio of 1/80 was used, mTG (specific activity: 31 U/mg) was added to reach an Enzyme/Substrate (*w*/*w*) ratio of 1/3.5 for chCE7degl and 1/6 for chCE7agl. The enzymatic conjugation of (DOTA)_1_-, (DOTA)_3_- or (DOTA)_5_-decalysine constructs to chCE7_agl_ led to a single species of immunoconjugates, with a precise and predictable BFCA/mAb ratio, comprising of two, six or ten DOTA-chelators, respectively. As expected, the conjugation occurred via Gln295/297 residues, but the addition of only one (DOTA)_n_-decalysine construct per each HC was observed (Table 5, panel B) as a result of possible steric influences. Radiolabeling was carried out at 37 °C incubating the immunoconjugates with ^177^LuCl_3_ in acetate buffer (pH 5.0) for 2.5 h. The increase in the number of DOTA-chelators linked to the Lys-substrate was attended by an increasing specific activity of the ^177^Lu-tagged immunoconjugates. Data from biological assays showed that the increased number of metal chelators was not counteracted by the reduction of the immunoreactivity; actually all immunoconjugates exhibited excellent biodistribution profiles characterized by high and specific tumor uptake after 24 h post injection in mice bearing human SKOV3ip ovarian cancer xenografts and good pharmacokinetics that changed depending on the numbers of DOTA moieties coupled to the decalysine peptide [122].

Taking advantage from these results, authors extended the use of transglutaminase for the site-specific modification of c-myc-tagged antibody-like scaffolds (Table 5, panel C) [123]. The c-myc-tag peptide sequence (EQKLISEEDL) is widely used for the sensitive detection of recombinant proteins by a high-affinity anti-c-myc antibody. It is already integrated in the C-terminal portion of a broad variety of pro- and eukaryotic expression vectors. Hence, proteins and, in particular, antibody-like scaffolds that come from phage-display libraries are often c-myc-tagged [126]. Therefore, mTG was further exploited to form a stable isopeptide bond between the Gln on c-myc-tag placed on a Fab fragment, derived from the antifibroblast activation protein (α-FAP) antibody ESC11, selected as a model protein, and various primary amine-functionalized substrates including BFCAs for nuclear medicine purposes. Enzymatic functionalization of the recombinant protein was performed in PBS incubating ESC11-fab (6.6 μM) with the corresponding amine-functionalized chemical entity (80 molar equivalent) and mTG (6 U/mL) for 16 h at 37 °C. No conjugation was found after reaction of the corresponding untagged proteins with mTG and the amine-derivatized substrates, clearly indicating that mTG selectively targets the Gln of the c-myc-tag peptide (Table 5, panel C). Among the different tested polyazamacrocycles chelators, NH_2_-PEG_4_-DOTA gave the highest conjugation yield (∼80 %). ESC11-Fab-DOTA was labeled with ^111^InCl_3_ (16 h at 37 °C) to generate ^111^In-ESC11-Fab-DOTA, which was assessed on liposarcoma tumor-bearing mice. Subcutaneous tumors were successfully visualized by SPECT-CT imaging. Due to the flexibility of the ubiquitous DOTA chelator, therapeutic probes could easily be generated by using a suitable radionuclide [126].

Further advances of this method have been described by Schibli and coworkers in a recent preliminary report, where they utilized the stable solid-phase immobilization of MTG onto glass microbeads for the successive generation of site-specifically modified proteins (Table 5, panel D) [56]. Data reported clearly showed that immobilized mTG permits the efficient production of homogenous, stoichiometrically and site-specifically conjugated proteins, including antibody fragments (Fab, scFvs, c-myc-tag scFvs), as well as whole antibodies (in their deglycosylated form), through distinct glutamines and, unprecedentedly, also through lysines, with various bi-functional substrates. Immobilization of mTG was found to increase the enzyme’s activity and site selectivity. By using a lysine-mimicking substrate, a conjugation rate of ≥90–95% was attained within 30–90 min of incubation for antibody fragments, meanwhile for deglycosylated mAb ≥ 95% conversion was achieved after overnight incubation, whereas site-specific Lys conjugation of proteins was also achieved by using immobilized mTG and an improved Q-tag selected from a small library of Gln-containing peptides. Having established that both Gln and Lys conjugation are accessible with immobilized MTG, the method was utilized to generate/develop dual-site-specifically modified antibodies for multimodal applications (Table 5, panel D). Site-specifically conjugated mAb comprising of a moiety for click chemistry and a moiety for bioorthogonal chemistry was obtained by the additional clicking of a fluorescent probe and a metal chelator for radiolabeling (-DOTA-like chelator), laying the base for the design of dual labeled antibodies useful in multimodal imaging for noninvasive and intra-/post-operative imaging, for instance, as well as in theranostic applications for imaging and simultaneous therapy [56].

Recently, we have explored the feasibility of the transglutaminase method for the selective conjugation of a bis(thiosemicarbazone) (BTS) bifunctional chelator to Substance P (SP) undecapeptide (Table 5, panel E) [124]. Diacetyl-2-(N4-methyl-3-thiosemicarbazone)-3-(N4-amino-3-thiosemicarbazone chelator H_2_ATSM/A, was identified as effective chelator of ^99m^Tc(N) and ^64^Cu^2+^ ions, and, it was functionalized with 6-aminohexanoic acid (ε-Ahx) to generate a bifunctional lysine-mimicking substrate, H_2_ATSM/A-ε-Ahx, suitable for conjugation to Gln residues of SP via mTG. SP comprises of two Gln residues (Gln5 and Gln6) and one Lys residue with very low reactivity. According to the stability data collected for H_2_ATSM/A-ε-Ahx [124], to minimize the isomerization/degradation of the chelator, conjugation was performed in phosphate-citrate buffer (pH 6.0), incubating the reaction mixture at RT for 2 h. The SP/H_2_ATSM/A-ε-Ahx chelator molar ratio was 1/10, meanwhile mTG was added to reach an enzyme-to-substrate ratio of 1: 20 (*w*/*w*). Unfortunately, the scarce H_2_ATSM/A-ε-Ahx stability was reflected on the type of the conjugation products and on the conjugation yield (low). However, the desired mono-derivate H_2_ATSM/A-ε-Ahx-SP species was attained with the selective conjugation of BFCA at the level of Gln5 according to literature data that indicated Gln5 as the amino acid residue with higher reactivity to TGase compared to Gln6 [127,128]. The mono-derivative preserves the binding properties of the native peptide [129]. Labeling was efficiently performed with both ^99m^Tc and ^64^Cu radionuclides in mild reaction conditions (RCYs ≥ 98% by using 10^−6^ M of H_2_ATSM/A-ε-Ahx-SP). In spite of this, the SP-conjugated radioconstructs were found unstable in sera, thus no further in vitro and in vivo biological studies are reported [124].

Most recently, we have been investigating the possibility of extending the usage of the [^99m^Tc][Tc(N)(PNP)]^2+^strategy (PNP = water-soluble diphosphino-amine) to the labeling of temperature-sensitive biomolecules such as proteins and derivatives (*unpublished data*) [125]. In this our preliminary work, apo-form of myoglobin (apoMb) was selected as a model protein. It was pertinently derivatized via site-specific enzymatic reaction catalyzed by mTG, with the CGLG tetra-peptide as Lys-substrate, enabling the incorporation into the protein backbone of a chemical accessible Cys residue as BFCA of [^99m^Tc][Tc(N)(PNP)]-synthon (Table 5, panel F). An apoMb/tetra-peptide molar ratio of 1/30 and an enzyme/substrate of 1/50 (*w*/*w*) were used. Notably, apoMb contains two Gln residues (Gln91 and Gln152) that can be derivatized by mTG, thus leading to the generation of a bis-derivative adduct. Upon conjugation (37 °C for 4 h), analyses revealed the formation of a main product identified as the mono-conjugated apoMb modified site specifically at the level of the Gln91 residue, and of only a minimal amount of the bis-derivative one modified at both Gln91 and Gln152 residues as result of the high substrate specificity of mTG (Table 4) [48]. Radiosynthesis was efficiently conducted in mild reaction conditions, at neutral pH, by incubating the [^99m^Tc][Tc≡N]^2+^_int_ precursor with the wsPNP ligand and the Cys~apoMb adduct (10^−5^ M) at RT for 30 min. The radiochemical yield of the ^99m^Tc-Cys-apoMb is 95%. The radiolabeled construct is highly stable under all investigated conditions, suggesting a role of the [^99m^Tc][Tc(N)(PNP)]-technology in the labeling of (temperature)-sensitive biomolecules for SPECT imaging.

The high regioselectivity of mTG has been recently questioned by Cornelissen et al. Authors assessed the mTG-mediated strategy in the generation of ^89^Zr-radiolabeled DFO-mAb conjugates with enhanced homogeneity, revealing unappreciated limits of Q-tag site selectivity at positions likely to directly impair function [130]. In this work, the combined PNGase and mTG methods were utilized to produce conjugates of anti Her2 Herceptin (Her). Reaction was performed by incubating the deglycosylated Her antibody (dgHer) with a pertinent primary amine (azido amide H_2_N–CH_2_CH_2_–(OCH_2_CH_2_)_2_–N_3_), as co-substrate of mTG to incorporate an azide residue at the target site (Q298 in Her) for subsequent clicking with strained alkynes. Characterization by traditional analysis modes (LC-MS, SDS-Page under reducing conditions) proved consistent with the high site-specific modification of Her, as previously indicated [45,55]. However, high resolution native MS analysis disclosed mixtures of non-homogeneous Ab species with varied conjugation grades up to three payloads plus unreacted dgHer, and indicates only 70–80% of functionalization at Q298 side chain, in competition with modification at another site, such as Q3 critically close to the CDR1 region, yielding a low percent of three-derivative. In spite of this, the authors were able to generate quite homogeneous mixtures with a substrate/mAb stoichiometry of 2:1 confirmed also by native MS, suitable for further radiolabeling studies. Radiosynthesis was efficiently performed in mild conditions, via DFO-chelation with ^89^Zr, yielding ^89^Zr-labeled dg-Her variants with RCY ranging from 94 to 96%. RICs were assessed in vitro and in vivo and compared with ^89^Zr–Herceptin conjugates obtained through conventional, random Lys-directed modification. Prior in vivo comparisons displayed that there are no differences between random attachment methods and more selective methods. This work highlights as the usage of appropriate and multiple analyses methods can provide more precision and accuracy in the characterization of the final adducts.

### 6.2. Sortase A-Mediated Conjugation in MI

SortaseA (SrtA) enzyme from *Staphylococcus aureus* has been extensively used for protein engineering and antibody modification to create a number of homogeneous fluorophore labeled antibodies and antibody fragments and also found its role in MI field. Sortase A has been used to modify single-chain (scFv) and single-domain antibody (sdAb; nanobody) fragments bearing the sortag recognition motif on their C-termini with several different (poly)Gly-functionalized BFCA, including CHX-A″-DTPA, NOTA, and sarcophagine (Table 6) [131,132,133,134,135,136,137,138]. In 2014, Donnelly and coworkers described the use of SrtA to site specifically append a (Gly)_n_-tagged (n = 1–3) MeCOSar sarcophagine chelator for ^64^Cu^2+^ ions to an anti-LIBS scFv bearing a C-terminal LPETGG-FLAG tag. Then, they described a creative SrtA-based conjugation strategy based on a two steps modular approach for scFvs modification: the first step uses the SrtA for the site-specific incorporation of orthogonal alkyne functional groups into scFvs to enable further modification by cycloaddition click reaction (Table 6 panel A). Conjugation was performed at 37 °C for 5 h, and 87–89% conversion was achieved by reacting the scFv anti-LIBS-LPETGG-FLAG tag with glycine containing substrates and SrtA in a molar ratio of 3:1:3 in sortase reaction buffer (50mM tris, 150 mM NaCl, 0.5 mM CaCl_2_ pH 8.0). The second step of the approach involved the azide–alkyne cycloaddition click reaction to allow for the selective and reproducible attachment of fluorophores or BFCAs suited for radiometals.

**Table 6:** Overview of Sortase A mediated conjugation in radiopharmaceutical applications.

Biomolecules and Strategy		Bi-Functional Substrate	Radionuclide	Ref.
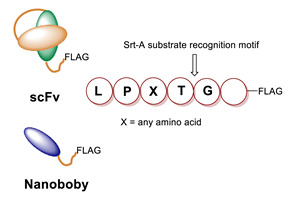	**A**	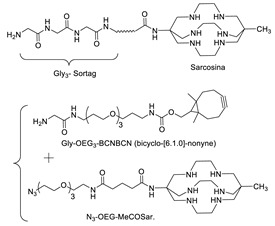	^64^Cu	[131,132]
	**B**	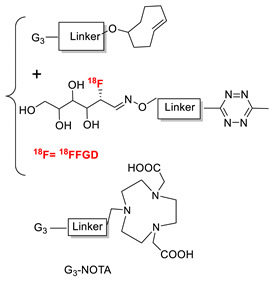	^18^F; ^64^Cu	[133,134,135]
	**C**	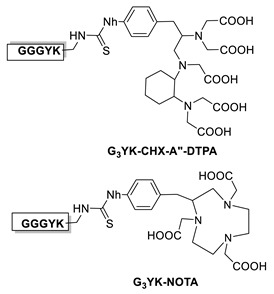	^111^In; ^68^Ga	[136,137]

Alongside, Ploegh et al. in an independent paper described a strategy based on a two steps modular process to label with 2-[^18^F]-fluorodeoxy-D-glucose (FDG) and ^64^Cu sdAb fragments to explore the possibility of truck immune responses [133,134]. SrtA-mediated reaction was used to include a trans-cyclooctene (TCO) functionalized short peptide on the protein of interest (scFv VHH7) producing a reactive TCO labeled protein. Then, it was reacted (15–20 min, RT) with a [^18^F]-FDG-tagged Tz to generate, according to the prelabeling method, the final RIC (RCY = 25%). This technique is further applicable to install a selected functionality (such as NOTA) to any other suitably modified biomolecule (Table 6, panel B). In the following work, the authors utilized SrtA to develop camelid single-domain antibody fragments site specifically tagged with two handles: one for the introduction of a fluorophore or a radionuclide (eg ^18^F) for different imaging modalities, and the second for further modification of the fragment with a PEG moiety or a second antibody fragment to tune its circulatory half-life or its avidity [135].

More recently, Devoogdt and coworkers also explored the protein ligation capacity of SrtA to modify sdAb (Her2 targeting 2Rs15d; Bcll10 as control) [136]. Nanobodies were expressed with a C-terminal sortag LPETG followed by a His-tag to facilitate the purification process. The versatility of the approach was demonstrated by conjugating independently three different imaging probes properly modified with the GGGYK pentapeptide: the chelating agents CHX-A”-DTPA and NOTA for SPECT with ^111^In and PET with ^68^Ga, respectively, and the fluorescent dye Cy5 for fluorescence reflectance imaging (Table 6 panel C). The pentapeptide served as the base for the imaging probes. It contained an N-terminal triglycine to fulfill the nucleophilic attack in the enzymatic reaction and a Lys residue as the anchoring site for the BFCAs and for the fluorescent dye Cy5. An overnight reaction with a molar ratio sdAb:SrtA:GGG-substrate 1:3:30 was found optimal to attain a sdAb substrate consumption above 75%. Upon the purification process, homogeneous single-conjugated species populations were obtained in high yield (30–50%) [138]. Radioimmunoconjugates were efficiently generated. The enzymatic conjugation did not affect the affinity of the tracers for the molecular target (human epidermal growth factor receptor 2, HER2) both in vitro and in vivo. Imaging of tumor BT474M1 xenografts revealed the ability of the conjugates to visualize the tumor and a good contrast as early as 1 h post-injection. These outcomes were also confirmed by ex vivo biodistribution studies of ^111^In-CHX-A”-DTPA-2Rs15d and ^68^Ga-NOTA-2Rs15d. Analyses displayed a high and specific tumor uptake at 90 min post-injection and low background in all other non-targeted organs, except the kidneys (excretion organs).

Thus far, authors exploited the above-mentioned approach for the site-specific conjugation of the NOTA chelator to a nanobody to assess human PDL1 expression by PET [137]. The coupling was performed by the overnight incubation of SrtA enzyme with a hPD-L1 sdAb containing a sortag at the C-terminus and the GGGYK-NHCS-Bn-NOTA construct characterized by the triglycine sequence, acting as a nucleophile attacking the sdAb-SrtA intermediate. Site-Specific sdAb functionalization was performed by incubating (16 h at 37 °C) the hPD-L1-sortag-His6-tag nanobody in Tris-buffered Saline (TBS; pH 7.0) with GGGYK-NHCS-Bn-NOTA and Srt-A enzyme in 1:20:2 molar ratios (56%yield). The site-specific conjugation resulted in a homogenous product of sdAb coupled to one NOTA chelator. Radiolabeling with ^68^Ga was efficiently conducted at RT within 10 min (RCP 95%). In vitro and in vivo evaluations were also reported, and the collected data have been compared with the biological profile of the corresponding RIC obtained by a conventional chemical approach using random conjugation to Lys residues through the protein, whose resulted in a mixture mainly containing sdAbs with zero or one NOTA chelator, as determined by ESI-Q-ToF-MS analysis.

Notably, sdAb contains in total four lysines, including a Lys residue located in one of its binding regions. It is unknown which Lys or how many lysines will be coupled to NOTA after a random coupling, and thereby this strategy could affect the biomolecule’s binding potential. Biological studies of the site-specific conjugate showed excellent targeting properties with no off-target accumulation. However, the tumor uptake of the randomly radiolabeled sdAb is as good as the site-specifically radiolabeled one [137]. Together with the afore mentioned reports, this study demonstrates the versatility and efficacy of SrtA to produce imaging tracers for multiple non-invasive in vivo imaging modalities.

### 6.3. Galactosyltransferase-Mediated Conjugation in MI 

In 2013, Zeglis et al. combined bioorthogonal click chemistry and the polypeptide-α-N Acetylgalactosaminyltransferase for the site-specific modification of Fc domains of antibodies (Table 7, panel A) [106]. The prostate specific membrane antigen-targeting antibody J591 was used as the model protein. It was derivatized with the DFO chelator for labeling with ^89^Zr by adopting a multistep approach which requires: (1) the enzymatic removal by β-1,4-galactosidase action (37 °C overnight; NaH_2_PO_4_ 50 mM, pH 6.0) of the terminal galactose residues from the biantennary complex-type oligosaccharide on the Fc domain of mAb, to expose the terminal GlcNAc residues; (2) the incorporation of an azide-modified galactosamine (GalNAz; *N*-azidoacetylgalactosamine) using a mutant, substrate-permissive 1,4,-galactosyltransferase Gal-1T1(Y289L) to afford, after overnight incubation at 30 °C, the modified mAb bearing bioorthogonal azido groups, N_3_-mAb; (3) the click conjugation (overnight incubation at 25 °C) of dibenzocyclooctyne-bearing BFCA (DIBO−DFO) to the N_3_-mAb following the strengthened copper-free SPAAC method. Purification via size exclusion chromatography yielded the site-selectively modified DFO-DIBO-mAb in 49 ± 5% yield, and the number of chelates appended per mAb was 2.8 ± 0.2 on a maximum number of chelators for mAb of four; (4) the labeling of DFO-J591 adduct with ^89^Zr. In vitro assessment and in vivo biodistributions of ^89^Zr-tagged DFO-J591 were performed on the appropriated human prostate cancer model and compared with the biological profiles of the corresponding RIC obtained by random chemical conjugation, for which the number of DFO-NCS appended per mAb was 3.1 ± 0.5. The ^89^Zr−DFO−J591 was found to be as stable as the conventionally prepared ^89^Zr−DFO−NCS−J591 (>96% after 7 days incubation in serum); however, in vitro and in vivo studies demonstrated that both RICs performed in a nearly identical behavior [106]. 

By using the above chemo-enzymatic approach, the same research group developed a site-specifically dual-labeled (PET−optical imaging [OI]) mAb (huA33, colorectal cancer targeting mAb) [107]. In this case, the click-chemistry reaction was conducted by reacting the site-specific modified N_3_-huA33 with a mixture of DFO−DIBO (for ^89^Zr) and Alexa Fluor 680-DIBO (near infrared fluorescent dye), affording a hybrid imaging agent with different chelator-to-dye-to-mAb ratios (from 1:1.3:1 to 2.9:0.5:1) (Table 7 panel B). N_3_-huA33 construct was also pertinently exploited to create a site-specifically labeled huA33-trans-cyclooctene immunoconjugate (^ss^huA33-PEG_12_-TCO) by reacting the azide-modified mAb with a dibenzocyclooctyne-bearing variant of TCO (DIBO-PEG_12_-TCO). It was utilized to develop a pre-targeted PET imaging strategy based on the rapid and bioorthogonal click reaction between a ^64^Cu-labeled tetrazine radioligand (^64^Cu-Tz-SarAr) and the prepared ^ss^huA33-PEG_12_-TCO (Table 7 panel C) [139,140,141]. Then, they also used the same approach to prepare a PET−OI bimodal immunoconjugate based on pancreatic ductal adenocarcinoma (PDAC) targeting antibody 5B1(Table 7, panel B) [142] and a dual site-specific immunoconjugates based on a HER2 targeting trastuzumab carrying both a monomethyl auristatin E (MMAE) toxin and a ^89^Zr-DFO payload for PET imaging. The tumor targeting and therapeutic efficacy of the ^89^Zr-trastuzumab-MMAE immunoconjugate were validated in vivo using a murine model of HER2-expressing breast cancer (Table 7 panel D) [143]. 

Homogenous site-specific immunoconjugates may disclose superior pharmacological properties than that of the corresponding poorly defined and heterogeneous biologics produced via random conjugation methodologies. In a very recent work, Kristensen et al. reported the effect of site-specific labeling on the stability, immuno-reactivity, and tumor-targeting properties of trastuzumab and they compared it to conventional random labeling on Lys residues in a HER2-positive xenograft mouse model [144]. Site-specific chemo-enzymatic labeling of trastuzumab minimizes the impact of the DFO chelator on immuno-reactivity, stability, and biodistribution. Indeed, the site-specifically radiolabeled ^89^Zr-DFO-trastuzumab displayed good in vitro properties with increased stability and immuno-reactivity when compared to ^89^Zr-DFO-trastuzumab conjugated by conventional Lys chemistry. Furthermore, site-specific ^89^Zr-DFO-trastuzumab exhibited superior tumor-targeting properties in the SK-OV-3 model.

Likewise, Vivier et al. also explored in a comparative study the effects of the above chemo-enzymatic approach of site-specific bioconjugation on the in vivo performance of selected RICs (Table 7 panel E) [145]. The truncation of the heavy chain glycans of the RIC was expected to reduce its off-target uptake and increase the tumor accumulation [146]. It was found that this modification diminishes the binding of the immunoconjugates to the Fc-γ-receptor I (FcγRI). FcγRI is a member of the FcγR family that possesses high affinity for the Fc region of immunoglobulins. It is able to bind monomeric IgGs and it is expressed by immune cells and tissue-resident macrophages. FcγRI has been identified as a possible cause of the off-target accumulation of RICs. Besides, the affinity of Fc to FcγR would seem to be sensitive to the glycosylation state of mAb, although glycans are not directly involved in the interactions. However, deglycosylated mAbs have shown reduced binding affinity to FcγR and have been proved to possess improved in vivo performance [146]. Due to its clinical relevance, HER2 targeting pertuzumab was selected for the study and DFO-labeled pertuzumab immunoconjugates were prepared: one using traditional, random Lys bioconjugation methods (DFO^nss^pertuzumab, with an average of 1.4 ± 0.4 DFO per mAb) and two using the chemo-enzymatic protocols that alter the glycan biantennary chain: DFO^ss^pertuzumab-βGal with an average of 2.6 ± 0.1 DFO per mAb obtained by the action of β-1,4 glycosidase (vide supra) and DFO-^ss^pertuzumab-EndoS with an average of 1.3 ± 0.2 DFO per mAb produced by EndoS enzyme, which hydrolyzes the chitobiose core of the Asn297-linked glycans producing a partially deglycosylated mAb. The in vitro ability of the constructs to bind recombinant HER2 as well as human and mouse FcγRI were explored alongside with the in vivo performance in two different mouse models of HER2-expressing BT474 human breast cancer, one including athymic nude mice and the other humanized NSG (huNSG) mice. In vitro studies revealed that all three immunoconjugates bind HER2 as effectively as native pertuzumab. An abrogated binding affinity for huFcγRI of the deglycosylated DFO-^ss^pertuzumab-EndoS adduct compared to native pertuzumab DFO-^nss^pertuzumab and DFO-^ss^pertuzumab-βGal was confirmed.

PET imaging and biodistribution experiments in athymic nude mice bearing BT474 xenografts yielded no significant differences in the behavior of the non-site-specific and site-specific ^89^Zr-tagged mAbs. Nevertheless, experiments in tumor-bearing huNSG mice revealed that site-specific RICs produces higher tumor uptake and lower activity concentrations in non-target tissues (e.g., liver and spleen) than the non-site-specifically labeled counterpart, a phenomenon that may be due to the altered binding of the formers to huFcγRI [145]. Notably, ^89^Zr-DFO-^ss^pertuzumab-EndoS was the best performing adduct.

**Table 7:** Overview of galactosyltransferase mediated conjugation in radiopharmaceutical applications.

	Biomolecules and Strategy	Bi-Functional Substrate	Radionuclide	Ref.
**A**	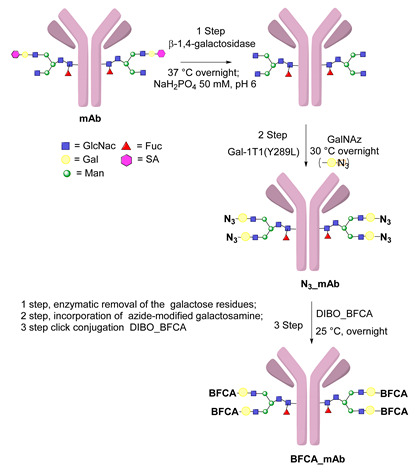	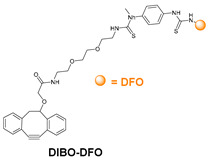	^89^Zr	[106]
**B**	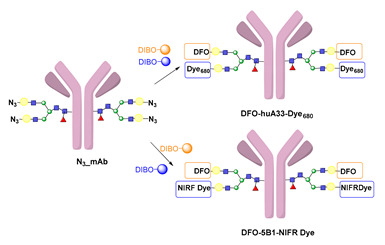	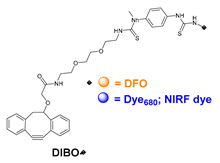	^89^Zr	[107,142]
**C**	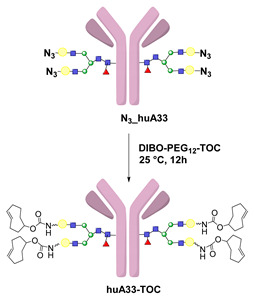	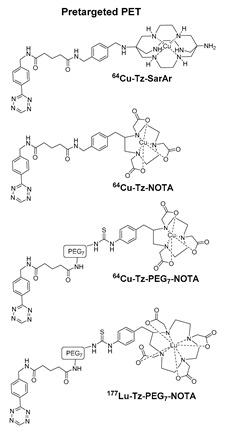	^64^Cu, ^177^Lu	[139,140,141]
**D**	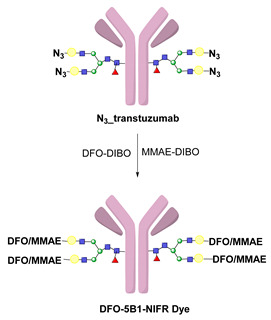	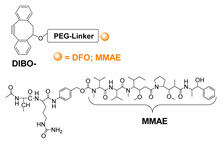	^89^Zr	[144,147]
**E**	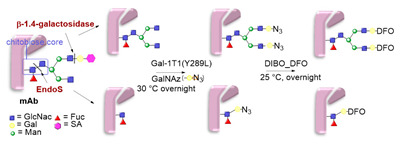	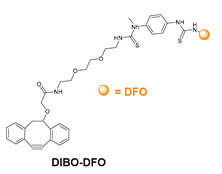	^89^Zr	[145,146]

### 6.4. Lipoic Acid Ligase-Mediated Conjugation in MI

Very recently, Drake et al. described the use of the lipoic acid ligase (LplA) for the site-specific coupling of ^18^F-labeled carboxylate substrates to biomolecules [113]. Site-specificity was achieved by introducing an LAP-peptide tag (Figure 5) into the C-terminal portion of Fab by standard cloning techniques. In this work, authors picked 8-[^18^F]-Fluorooctanoic acid ([^18^F]-FA) and 7-(4-[^18^F] fluorophenyl)-7-oxyheptanoic acid ([^18^F] FPOA) as prosthetic groups since octanoic acid is a known substrate of LplA, and the recombinant human Fab antibody fragment 2G10 for proof of concept (Table 8). Enzyme-mediated reactions were fast (10–15 min) and highly efficient (high conjugation yields∼95%) under mild conditions (aqueous reaction, pH 7.4, 30 °C) with very small amounts of the Fab protein precursor (10 nmol) thanks to the low KM (13.3 μM) of LplA. This feature represents an important aspect of LplA usage because it can provide RIC with high specific activity that is crucial for the visualization of low-density in vivo targets (vide supra) [35]. LplA has clearly the potential for mediating the site-specific radiolabeling of biomolecules, but its reliability needs to be confirmed by further experiments.

**Table 8:** Overview of lipoic acid ligase mediated conjugation in radiopharmaceutical applications.

Biomolecules and Strategy	Bi-Functional Substrate	Ref.
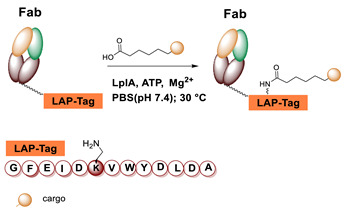	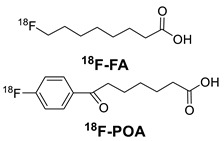	[113]

## 7. Concluding Remarks

The use of enzymes as highly efficient and site-specific alternatives to the classical random chemical approaches has been gaining increasing interest in biotechnology and it is also finding its role in radiopharmaceutical applications for developing site-specific radiolabeling and highly homogeneous RIC. A precise control of the number and location of payloads on the protein should really result in better-defined and more efficient immunoconjugates and more easily reproducible procedures and may facilitate the approval process. In general, the enzymatic derivatization of proteins offers important advantages such as the use of aqueous mild reaction conditions that are required to preserve the integrity of the biomolecules as well as of BFCA and prosthetic groups. They can be efficiently used to selectively modify proteins and derivatives through one step protocols or stepwise procedures combining mild enzymatic methods with high-yielding bioorthogonal click reactions (vide supra). The inclusion of groups suitable for bioorthogonal reactions enables a modular approach with the potential for adding multiple functional moieties without any apparent important effect on the mAb function. From the studies conducted so far on different enzymatic approaches, it is not obvious to define the best methodology for the design of homogeneous RICs, also because they are “biomolecule and radionuclide dependent” and the optimization of the procedure is in some cases necessary. The four enzymatic approaches considered in this review all proved useful for the radiolabeling of mAb and their truncated derivatives. However, each approach possesses intrinsic pros and cons that are summarized in Table 9.

Site-specific conjugations are also expected to improve the stability and pharmacological profiles of RICs in respect to biomolecules modified using traditional chemical approaches. Indeed, heterogeneous conjugation may result in a high cargo loading that can be responsible for their impaired immunoreactivity, low stability, and suboptimal pharmacokinetic profiles. Nevertheless, if on the one hand, animal studies have shown that site-specifically labeled RICs may feature superior in vivo behavior compared to their randomly constructed counterparts, on the other there is no irrefutable evidence of the added value of site-specific conjugation in comparison to random labeling. In most of the studies comparing both approaches, the site-specifically modified biomolecules seem to not outperform the conjugates prepared by conventional routes. This behavior may be due to the specific biomolecules and/or mouse model used, thus additional studies are urgent needed to support, confirm, or refute that site-specific modification in general will improve nuclear MI with mAb and related.

Although the benefits of site-specific labeling seem to be antibody-dependent, a systematic evaluation of the in vivo data of site-specifically derivatized mAbs for which the partial or complete removal of heavy chain glycans is required before enzymatic conjugation reactions, such as in transglutaminase and galactosyltransferase mediate methodologies, show a clear and significant improvement of the in vivo performance of RICs [45,144,145]. In all cases, the distribution profiles were characterized by higher target uptake and reduced off-target accumulation with respect to those of the full glycosylated and random derivatized versions. This happening finds its explanation in the relationship between deglycosylation and FcγR and in particular in the reduced affinity of the formers for FcγRI [144,145,146]. Despite the high risk of aggregation when we modify the mAb’s glycosylation state, these findings suggest that deglycosylation represents an inexpensive, efficient, and versatile approach that can be coupled with the advantages of different site-specific enzymatic strategies for the creation of immune-silent RICs with improved in vivo performance and images quality.

The examples presented here demonstrate the versatility and utility of site-specific enzyme-mediated bioconjugation to improve the efficiency and potential of protein-based radiolabeled products. Most of them are already tested in preclinical studies. However, further efforts concerning scale-up and cost minimization are necessary to realize the entire potential of site-specific protein modification in the clinic. The precise control provided by these bioconjugation methods can be used to identify criteria for selecting modification sites on mAbs and relates as well as determining generalizable design principles to maximize stability and circulation properties, biological activity, and targeted cellular uptake. Future developments in this area will also depend on the identification of new protein scaffolds that have appropriate characteristics in terms of stability, high target affinity, selectivity, rapid clearance, and ease of labeling, etc., as cancer targeting imaging agents. Moreover, by considering the current increased attention to the imaging of inflammatory responses by the direct targeting of T-cells, it is also expected that RICs and relates will be used to specifically target antigens of pathogenic bacteria and viruses, and thus permit the development of new pathogen-specific tracers to discriminate between infectious and sterile sites of inflammation.

## Figures and Tables

**Table 9:** Pros and cons of the discussed enzyme-based approaches.

Enzyme	Substrates	Biomolecules on MI	Pros	Cons
**mTG**	- *Acyl donor*: Gln residues of the protein; Q-tag inserted at any site - *Acyl acceptor*: Lys residues of the protein; K-tag inserted at any site; primary ammine	Wild type molecules ranging from whole mAbs to peptides	- homogeneous adducts with a precise control of the number and the location of the payloads - formation of catabolically stable isopeptide bonds - simple 5-aminopentyl groups can be used as lysine surrogates - no special antibody engineering is required - mTG immobilization increases its reactivity and permits a straightforward purification of IC - minimal off-target reactivity - one-step reaction with the cargos - low-cost enzyme	- cross-linked oligomers can be generated in the presence of reactive Gln and Lys residues in the same protein substrate - multiples sites of derivatization are possible - long reaction time for heavy proteins can be required
**SortA**	- *Acyl donor:* LPXTG sequence inserted at the C-terminus or internal sites - *Acyl acceptor:* (Gly)_n_ inserted at the N-terminus	Full-length mAbs and their fragments	- site-specific with no risk of impairing mAb reactivity - one-step reaction with the cargos	- need of mAb engineering - reversible reaction which requires a high molar excess of SrtA and the nucleophile over the LPXTG-substrate - expensive
**Galactosidase** and **GalT(Y289L)**	- Biantennary complex-type oligosaccharide of mAbs - Galactose modified with a functional group for click chemistry	Full-length mAbs with pendant sugar chains	- site-specific with no risk of impairing mAb reactivity - no antibody engineering is required - glycans can be manipulated without altering the polypeptide chain - the bi-antennary nature of the two oligosaccharide chains allows for at least two and as many as four conjugation events per mAb - the labeling sites are easily and rapidly characterized - bioorthogonal click ligation - minimal off-target reactivity	- hardworking (four-step) and time-consuming approach: various buffer exchanges by microspin columns are needed and long incubation times - inability to vary the conjugation site beyond glicans - usefulness limited to the whole mAb with pendant sugar chain and glycosylated proteins
**LplA**	- *Acyl donor*: lipoic acid and its analogues - *Acyl acceptor*: LAP-peptide inserted at any site	Full-length mAbs and their fragments	- site-selective with no risk of impairing protein activity - fast one step labeling - high catalytic efficiency - formation of catabolically stable isopeptide bonds - bioorthogonal click ligation - minimal off-target reactivity	- need of engineered proteins and lipoate analogues

## Data Availability

No new data were created or analyzed in this study. Data sharing is not applicable to this article.

## Abbreviations

3p-C-DEPA: 2-[(carboxymethyl)]-[5-(4-nitrophenyl-1-[4,7,10-tris-(carboxymethyl)-1,4,7,10-tetraazacyclododecan-1-yl]pentan-2-yl)-amino]acetic acid; ADC, Antibody-drug conjugates; BFCA, Bifunctional chelating agents; CD20, B-cell differentiation antigen; CH2, constant heavy chain 2; CPTA, 4-(1,4,8,11-tetraazacyclotetradec-1-yl) methyl benzoic acid; CuAAC, copper-catalyzed azide-alkyne reaction; cysteine, Cys; DBCO, Dibenzocyclooctynes; DFO, Deferoxamine; Diamsar/SarAr, 1-*N*-(4-Aminobenzyl)-3,6,10,13,16,19-hexaazabicyclo[6.6.6]-eicosane-1,8-diamine; DOTA, 1,4,7,10-tetraazacyclododecane-1,4,7,10-tetraacetic acid; EA, ethyl azide; EGFR, Epidermal growth factor receptor; EndoS, endoglycosidase; EPR, enhanced permeability and retention; Fc, fragment crystallizable region; GalT (Y289L), mutant of β-1,4–galactosyltransferase; GlaNAc, N-acetylgalactosamine; Gln, glutamine; HER2, human epidermal growth factor receptor 2; HYNIC, 6-hydrazinopyridine-3-carboxylic acid; IA, injected activity; IEDDA, inverse electron-demand Diels-Alder reaction; IgG, Immunoglobulin G; LA, α-lactalbumin; LplA, lipoic acid ligase; LC, Light chain; Lys, lysine; mAb, monoclonal antibody; Mb, myoglobin; MI, molecular imaging; MS, mass spectrometry: mTG, Microbial transglutaminase; N_3_S, mercaptoacetyltriglycine; NOTA, 1,4,7-triazacyclononane-1,4,7-triacetic acid; OI, Optical imaging; PET, Positron emission tomography; PNGase F, N-glycosidase F; RIC, radioimmunoconjugate; RIT, radioimmunotherapy; RTX, rituximab antibody; scFv, single chain variable fragment; sdAb, single-domain antibody; SKOV3, ovarian adenocarcinoma cell line; SP, substance P; SPAAC, strain-promoted azide-alkyne cycloaddition; SPECT, single-photon emission computed tomography; SrtA, Sortase A; TCO, trans-cyclooctene; TGase, Transglutaminase, Tz, tetrazine.
